# A Family of Water‐Immiscible, Dipolar Aprotic, Diamide Solvents from Succinic Acid

**DOI:** 10.1002/cssc.202000462

**Published:** 2020-05-11

**Authors:** Fergal P. Byrne, Clara M. Nussbaumer, Elise J. Savin, Roxana A. Milescu, Con R. McElroy, James H. Clark, Barbara M. A. van Vugt‐Lussenburg, Bart van der Burg, Marie Y. Meima, Harrie E. Buist, E. Dinant Kroese, Andrew J. Hunt, Thomas J. Farmer

**Affiliations:** ^1^ Department of Chemistry University of York York YO10 5DD UK; ^2^ BioDetection Systems BV Science Park 406 1098 XH Amsterdam The Netherlands; ^3^ TNO Utrechtseweg 48 3704 HE Zeist The Netherlands; ^4^ Department of Chemistry and Centre of Excellence for Innovation in Chemistry Khon Kaen University Khon Kaen 40002 Thailand

**Keywords:** dipolar aprotic solvent, low-toxicity solvent, membranes, solvent effects, succindiamide

## Abstract

Three dipolar aprotic solvents were designed to possess high dipolarity and low toxicity: *N*,*N*,*N*′,*N*′‐tetrabutylsuccindiamide (TBSA), *N*,*N*′‐diethyl‐*N*,*N*′‐dibutylsuccindiamide (EBSA), and *N*,*N*′‐dimethyl‐*N*,*N*′‐dibutylsuccindiamide (MBSA). They were synthesized catalytically by using a K60 silica catalyst in a solventless system. Their water immiscibility stands out as an unusual and useful property for dipolar aprotic solvents. They were tested in a model Heck reaction, metal–organic framework syntheses, and a selection of polymer solubility experiments in which their performances were found to be comparable to traditional solvents. Furthermore, MBSA was found to be suitable for the production of an industrially relevant membrane from polyethersulfone. An integrated approach involving in silico analysis based on available experimental information, prediction model outcomes and read across data, as well as a panel of in vitro reporter gene assays covering a broad range of toxicological endpoints was used to assess toxicity. These in silico and in vitro tests suggested no alarming indications of toxicity in the new solvents.

## Graphical abstract

**Succinic success**: Three new dipolar aprotic solvents are synthesized catalytically from succinic acid. Interestingly, all are water immiscible, an unusual property for dipolar aprotic solvents. Tested in a Heck reaction, metal–organic framework synthesis, and membrane fabrication, they perform comparably to traditional dipolar aprotic solvents such as *N*‐methyl‐2‐pyrrolidone, and in silico and in vitro tests suggest no alarming indications of toxicity.

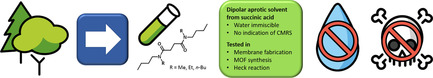

## Introduction

Dipolar aprotic solvents such as *N*‐methyl‐2‐pyrrolidone (NMP), *N*,*N*‐dimethylacetamide (DMAc), and *N*,*N*‐dimethylformamide (DMF) have many important functions throughout the chemical industry, such as in polymer production,^1^, ^2^, ^3^ organic synthesis,^4^, ^5^, ^6^, ^7^ graphene dispersion/exfoliation,^8^, ^9^ and metal–organic framework (MOF) synthesis.^10^ However, all are petroleum‐derived and suffer from high reprotoxicity.^11^, ^12^, ^13^, ^14^ As such, all are listed as substances of very high concern (SVHC) by the European Union's regulation REACH (Registration, Evaluation, Authorization, and Restriction of Chemicals),^15^ meaning alternatives are urgently needed.^16^

Progress has been made in this regard in recent years: new methods of solvent design have been developed,^17^, ^18^, ^19^ and new molecules have been discovered. Many ionic liquids can act both as solvents and catalyst for some synthetic applications.^20^, ^21^ Cyrene (levoglucosenone‐derived),^22^ propylene carbonate (carbon dioxide‐derived),^23^, ^24^, ^25^ and gamma‐valerolactone [hydroxymethylfurfural (HMF)‐derived]^26^, ^27^ have demonstrated dipolarity in a variety of applications. *N*‐Butylpyrrolidinone (NBP) is an amide solvent that has recently been developed by Eastman Chemical Company.^28^ It is structurally similar to NMP but contains an *n*‐butyl group instead of a methyl group, which results in non‐reprotoxicity.^29^ However, although the *n*‐butyl group eliminated reprotoxicity, it also reduced dipolarity compared with the traditional dipolar aprotic solvents.

The target of this work was to design a robust, biobased or bioderivable dipolar aprotic solvent that possesses high dipolarity and is non‐reprotoxic. As such, three new solvents have been proposed: *N*,*N*,*N*′,*N*′‐tetrabutylsuccindiamide (TBSA), *N*,*N*′‐diethyl‐*N*,*N*′‐dibutylsuccindiamide (EBSA), and *N*,*N*′‐dimethyl‐*N*,*N*′‐dibutylsuccindiamide (MBSA). They have been synthesized by using clean synthetic methodologies, including a reusable heterogeneous catalyst, and have been characterized for their physical and solubility properties. In addition, they have been tested in a model Heck reaction, metal–organic framework (MOF) synthesis, and solubility testing of industrially relevant polymers [polyvinylidene fluoride (PVDF), polyethersulfone (PES), and polyamide imides (PAIs)], in which they were shown to perform comparably to or in some cases better than traditional dipolar aprotic solvents. In other cases, interesting results were obtained owing to the water immiscibility of the succindiamides.

Finally, the effect of *n*‐butyl groups on diamides in terms of toxicity was examined. For this purpose, the compounds were analyzed by using an integrated testing strategy combining in silico predictions with in vitro reporter gene assays. The in silico prediction of toxicity of the compounds is a useful first step of toxicity analysis and focused on the human health endpoints decisive to authorization and restriction under REACH. This includes carcinogenicity (C), mutagenicity (M), and reproduction toxicity (R), and another health endpoint considered critical in this respect, skin sensitization (S). The CALUX® battery of in vitro reporter gene assays contains a range of specific tests that can be used for assessing chemical safety. It consists of 18 human cell‐based assays, each able to measure chemical interactions between a test compound and a specific nuclear receptor or cell signaling pathway.^30^ The use of these contrasting but complementary screening approaches aims to generate a more robust assessment of potential safety issues.

## Results and Discussion

### Solvent design

**Figure 1:**
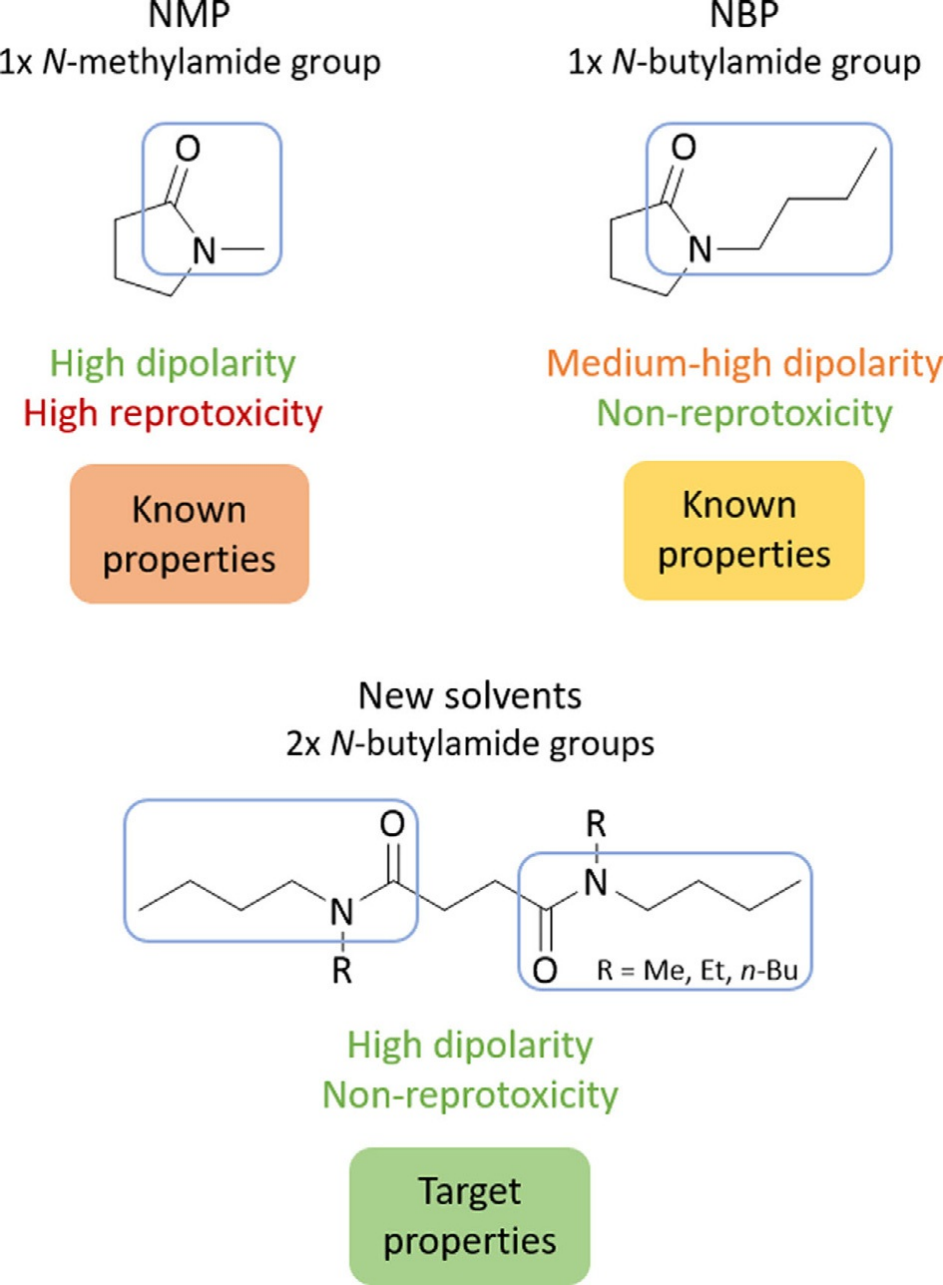
Hypothesized effect on polarity and toxicity of having two *n*‐butylamide groups on one molecule.

Inspired by NBP′s lower reprotoxicity compared with NMP,^28^ a range of similarly non‐reprotoxic, but more polar, molecules were sought. Three molecules were designed that targeted these solvent properties. It is not clear why, but because the *n*‐butylamide group on NBP is the only structural difference between NBP and NMP, it is this functionality that reduces reprotoxicity compared with the methylamide group of NMP. However, the consequence of the *n*‐butylamide group is an undesired lower dipolarity compared with traditional dipolar aprotic solvents. Therefore, it was hypothesized that by generating molecules that contain two *n*‐butylamide groups (*N*,*N*′‐dibutyldiamide), a combination of low reprotoxicity and high polarity could be achieved (Figure 1).

Succinic acid is one of the top value‐added chemicals from biomass proposed by the US Department of Energy (DOE) in 2004.^31^ Since then, it has been established as one of the most promising biobased platform chemicals^32^ with several companies targeting its commercialization.^33^, ^34^ Succinic acid can be produced either by the fermentation of sugars or by the oxidation of levulinic acid.^35^ Being a 1,4‐diacid, it was identified as an ideal chassis onto which *N*,*N*′‐dibutyldiamides can be built by reacting with alkylbutylamines (Figure 1). In addition, alkylbutylamines can be easily produced from biomass by the amination of bio‐butanol, bio‐ethanol, and bio‐methanol.

### Synthesis of *N*,*N*′‐dialkyldibutylsuccindiamides

**Scheme 1:**
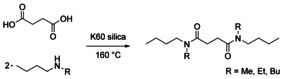
Syntheses of *N*,*N*′‐dialkyldibutylsuccindiamides from succinic acid.

The three new *N*,*N*′‐dialkyldibutylsuccindiamides were first synthesized by using succinyl chloride and the corresponding secondary amine as a proof of concept and to measure solvent properties (Table 1, entries 5–7). Upon confirmation that the solvents were indeed dipolar, the synthesis was attempted by the amidation of succinic acid with the corresponding secondary amines (Scheme 1). K60 silica calcined at 700 °C (K60‐700) has previously been demonstrated to catalyze the amidation of carboxylic acids with amines.^36^ K60‐700 is a robust solid catalyst, which is easy to produce, non‐corrosive, and can be recovered from the reaction mixture and reused after calcination again at 700 °C.^36^ As such, it was employed in the production of the new amides.

**Table 1:** Reaction yields for the synthesis of *N*,*N*′‐dialkyldibutylsuccindiamides.

Entry	Starting material	Product	Yield [%]
1	succinic acid	TBSA	45^[a]^
2	succinic acid	EBSA	31^[b]^
3	succinic acid	MBSA	<10^[c]^
4	succinic acid	MBSA	53^[d]^
5	succinyl chloride	TBSA	63^[e]^
6	succinyl chloride	EBSA	82^[e]^
7	succinyl chloride	MBSA	70^[e]^

[a] Open system, reflux conditions (≈160 °C), 18 h. [b] Open system, reflux conditions (≈110 °C), 18 h. [c] Open system, reflux conditions (≈90 °C), 18 h. [d] Closed system, increased pressure (180 °C), 18 h. [e] N_2_ flow, no temperature control (<35 °C), 18 h, CH_2_Cl_2_ solvent. See the Supporting Information for detailed experimental procedures.

The reactions were performed in a solventless system, with the amines being used in a large excess (15:1 molar ratio, amine/succinic acid). Owing to the higher boiling points of dibutylamine (160 °C) and ethylbutylamine (109 °C) compared with methylbutylamine, higher reflux temperatures could be obtained. As such, the synthesis of the corresponding amides, TBSA and EBSA, could be performed under reflux and atmospheric pressure, with yields of 31 and 45 %, respectively (Table 1, entries 1 and 2). The lower boiling point of methylbutylamine (90 °C) meant that the synthesis of MBSA only achieved a very low yield after 18 h (<10 %; Table 1, entry 3). As such, the reaction was instead performed in a closed system under elevated pressure to allow higher temperatures to be reached. This was achieved when the reaction was performed at 180 °C, with a yield of 53 % being obtained (Table 1, entry 4). Although the yields are moderate, unreacted starting material can be easily separated by using Kugelrohr short‐path distillation at 160 °C and 1 mbar and recycled back into the system for reuse. A small amount of the cyclic imide was produced as a side product in all cases, but this was also easily removed by distillation and can be recycled back into the system to undergo secondary amidation.

This process has the potential to be performed in continuous flow. For a flow process to be possible, both reactants (acid and amine) must be in the liquid phase because the K60 silica catalyst is a solid. However, succinic acid is a solid and not soluble in the amines. As such, potential for the succindiamide solvents to be used as the solvent in their own synthesis was examined. First, the solubility of succinic acid in the corresponding succindiamide was examined. It was found that 10 wt % succinic acid was soluble in MBSA at room temperature, allowing such a flow process to be investigated. However, succinic acid was largely insoluble in EBSA and TBSA. Succinic anhydride was then examined as an alternative to succinic acid and was found to be soluble in each of the succindiamides at 10 wt %. Succinic anhydride provides the added benefit of being reactive with the amines, forming the succinamic acid (acid‐amide), at room temperature without the need for a catalyst. Succinamic acid can potentially react with another equivalent of amine in the same conditions to produce the succindiamides. A full investigation into the flow synthesis of the new solvents is ongoing.

### Characterization of new solvents

Solvents properties are shown in Table 2. The boiling points of the succindiamides are higher than the traditional dipolar aprotics, being distilled under vacuum at 160 °C, whereas their melting points are significantly lower (−76 to −79 °C). Their densities are similar both to water and the traditional dipolar aprotic solvents.

**Table 2:** Properties of the new solvents in comparison with traditional solvents.

Solvent	*M*_w_ [g mol^−1^]	b.p. [°C]	m.p. [°C]	Density [g mL^−1^]	*V*_mol_ [cm^−3^ mol^−1^]	Log *P*_(o/w)_	*δ*_D_ [MPa^−0.5^]	*δ*_P_ [MPa^−0.5^]	*δ*_H_ [MPa^−0.5^]	*α*	*β*	π*	Water misc.	Hexane misc.
TBSA	340.55	>250^[a,b]^	−76^[e]^	0.96^[a]^	368.3^[f]^	3.77^[a]^	17.2^[f]^	9.0^[f]^	2.9^[f]^	0.00^[g]^	0.91^[h]^	0.63^[e]^	no^[a]^	yes^[a]^
EBSA	284.44	>250^[a,b]^	−76^[e]^	0.97^[a]^	299.6^[f]^	2.72^[a]^	17.2^[f]^	10.4^[f]^	3.3^[f]^	0.00^[g]^	0.91^[h]^	0.67^[e]^	no^[a]^	yes^[a]^
MBSA	252.36	>250^[a,b]^	−79^[e]^	0.99^[a]^	266.3^[f]^	1.65^[a]^	17.5^[f]^	11.0^[f]^	7.5^[f]^	0.00^[g]^	0.82^[h]^	0.78^[e]^	no^[a]^	yes^[a]^
NBP	141.21	241^[c]^	<−75^[c]^	0.96^[c]^	149.1^[f]^	0.99^[a]^	17.4^[f]^	6.7^[f]^	5.2^[f]^	0.00^[g]^	0.92^[h]^	0.77^[e]^	yes^[a]^	yes^[a]^
NMP	99.13	202^[d]^	−24^[d]^	1.03^[d]^	96.6^[f]^	−0.38^[d]^	18.0^[f]^	12.3^[f]^	7.2^[f]^	0.00^[g]^	0.75^[c]^	0.90^[c]^	yes^[a]^	no^[a]^
DMF	73.09	153^[d]^	−60^[d]^	0.94^[d]^	77.4^[f]^	−1.01^[d]^	17.4^[f]^	13.7^[f]^	11.3^[f]^	0.00^[c]^	0.71^[c]^	0.88^[c]^	yes^[a]^	no^[a]^
DMAc	87.12	166^[d]^	−20^[d]^	0.94^[d]^	93.0^[f]^	−0.77^[d]^	16.8^[f]^	11.5^[f]^	9.4^[f]^	0.00^[c]^	0.73^[c]^	0.85^[c]^	yes^[a]^	no^[a]^

[a] This work. [b] Distilled by Kugelrohr short‐path distillation at 160 °C and 1 mbar. [c] Sherwood et al.^25^ [d] Data obtained from PubChem. [e] Measured by differential scanning calorimetry. [f] Calculated by using HSPiP (version 5.1.08). [g] Assumed value. [h] This work, using *N*,*N*‐diethyl‐4‐nitroaniline and 4‐nitroaniline dyes. [i] This work, using *N*,*N*‐diethyl‐4‐nitroaniline dye.

NBP was found to be miscible with both water and *n*‐hexane . This is demonstrated by their octanol/water partition coefficients. The succindiamides have large, positive Log *P*_(o/w)_ values, meaning they favor the organic layer in an octanol/water biphasic system and are therefore more lipophilic.^37^ In contrast, the traditional dipolar aprotics have large, negative Log *P*_(o/w)_ values so are more hydrophilic. NBP displays intermediate properties, with a Log *P*_(o/w)_ of 0.99, meaning it prefers the organic phase but not enough to make it immiscible with water. Importantly, none of the succindiamide solvents have a Log *P*_(o/w)_ above 4, the value that has been set as a threshold for bioaccumulation in the environment.

The Hansen solubility parameters (HSP)^38^ and the Kamlet–Abboud–Taft (KAT) parameters of the new solvents were obtained.^39^, ^40^, ^41^ HSP characterizes solvents in terms of their dispersion forces (*δ*_D_), dipolarity (*δ*_P_), and hydrogen‐bonding ability (*δ*_H_). Higher values indicate stronger intermolecular interactions. KAT parameters provide similar information, but the dipolarity and polarizability (dispersion forces) are combined in one parameter (π*) whereas hydrogen‐bond‐donating (*α*) and ‐accepting ability (*β*) are separated. HSP values are predicted by using HSPiP software whereas KAT parameters are calculated by measuring the absorbance of dyes that are dissolved in the solvent.

Table 2 shows that the *δ*_D_ of each succindiamide is comparable to the traditional dipolar aprotics (17.2–17.5 MPa^−0.5^), likely owing to the common dominant amide functionality across all molecules. The *δ*_P_ of each candidate is in the range of 9.0–11.0 MPa^−0.5^, which is slightly lower than the traditional dipolar aprotics, the polarity of which ranges from 11.5–17.4 MPa^−0.5^, but higher than the other butylamide, NBP (6.7 MPa^−0.5^). MBSA provides the highest dipolarity of the succindiamides owing to its shorter alkyl chains, followed by EBSA and MBSA. Interestingly, each of the succindiamides, particularly TBSA and EBSA, possess far lower *δ*_H_ values than traditional dipolar aprotics. This is consistent with the Log *P*_(o/w)_ values and their immiscibility with water but miscibility with hexane, a very unusual property for polar solvents (Table 2).

Because none of the succindiamides are protic, *α* is 0.00 in all cases. The succindiamides, along with NBP, have higher *β* values than the methylamides NMP, DMF, and DMAc. MBSA, which is the least lipophilic of the succindiamides, falls in between the traditional butyl and methylamides in terms of *β*. Higher *β* values are owing to the greater electron donation of the butyl chains compared with the methyl chain. This conflicts with the HSP and Log *P*_(o/w)_ assessment of the succindiamides because a higher *β* would suggest an increased water miscibility. This suggests that either steric effects resulting from the long butyl chains block access to the amide functional groups, or that the average *β* across the larger succindiamide molecule is reduced compared with the traditional solvents.

The dipolarity/polarizability, π*, of each of the succindiamides is lower than traditional dipolar aprotics and closer to the butylamide, NBP. Because the KAT description of polarity is in contrast with the HSP description, several application tests were performed to assess the performance of the succindiamides in comparison to the traditional solvents.

### Application testing

To demonstrate the applicability of the new succindiamide solvents, they underwent a selection of solubility tests on industrially relevant polymers, PES membrane fabrication, a model Heck reaction,^24^ and as a solvent for MOF synthesis, which are described in the following sections.^42^

#### Industrially relevant polymer dissolution study

Polar aprotic solvents play a significant role in the production of a number of articles for which dissolution of specific polymers is required. Currently, these processes predominantly use the solvents NMP, DMAc, and DMF, and as such, alternatives are required. Three polymers are closely evaluated in this work: PAIs, PES, and PVDF. PAIs were first developed in the 1950s and became commercially available in the 1960s for use in injection molding.^43^ When requiring solvent application, they have been applied as a hard coating for kitchen appliances, a laminating resin, and most profusely as a wire enamel.^44^ The PAI utilized in this work is Torlon AI‐10, developed specifically for film‐forming applications.^45^

PES is a high‐temperature engineering thermoplastic principally used in formation of membranes owing to its excellent physical characteristics and the degree of control that can be achieved through modification of the casting system.^46^ The PES investigated in this work is Ultrason E3020.^47^ Finally, PVDF is a chemically and thermally stable but electronically active polymer.^48^ PVDF has many applications, including in membrane formation,^49^ medical sensors,^50^, ^51^ and as a binder in lithium‐ion batteries.^52^, ^53^ The grade of PVDF applied here is Solef 5130, which is widely utilized in battery production.^54^ All polymer dissolution studies were performed at 10 wt % loading (200 mg in 2 g of solvent) and heated to 80 °C with agitation by a magnetic stirrer bar, before being left to cool. MBSA, EBSA, TBSA, and NBP (Table 3) were used as the test solvents.

**Table 3:** Results of polymer dissolution at 10 wt % PVDF, PES, and PAI in MBSA, EBSA, TBSA, and NBP.

Solvent	PVDF^[a]^	PES^[a]^	PAI^[a]^
MBSA	soluble^[b]^	soluble	soluble
EBSA	soluble^[b]^	partially soluble	soluble^[c]^
TBSA	soluble^[b]^	insoluble	soluble^[c]^
NBP	soluble^[b]^	soluble	soluble

[a] Dissolution performed at 80 °C with agitation for 1 h. [b] Formed gel upon cooling. [c] Precipitation upon cooling.

All four solvents were able to dissolve PVDF at the dissolution temperature but produced a gel upon cooling. Hence, the stirrer bars could not be removed (Figure S1 in the Supporting Information). Only MBSA and NBP fully dissolved PES, partial dissolution was observed with EBSA, and no interaction was observed with TBSA. Finally, full dissolution of PAI was observed with MBSA and NBP, whereas TBSA and EBSA saw some polymer precipitate out of solution upon cooling. The results suggest these novel polar aprotics would all be suitable for use with PVDF and PAI, whereas MBSA could also be used in applications of PES. As such, membrane formation in a non‐solvent‐induced phase separation (NIPS) process was chosen as an application to test the performance of MBSA with PES.

#### PES membrane fabrication

The demand for clean water or controlled aqueous systems requires efficient treatment methods. Membrane filtration offers such a solution. Many polymers have been reported for membrane fabrication, such as cellulose acetate, PVDF, polyvinyl alcohol (PVA), and PES. PES has emerged as a particularly effective polymer for membrane fabrication because it offers high thermal, hydrolytic, and chemical stability.

Fabrication of PES membranes is traditionally done by using dipolar aprotic solvents such as NMP and DMSO. Because the solvent represents the largest contributor of waste in the production process, greener alternatives are required.^55^ Recently, a new green solvent, Cyrene, has been demonstrated to produce high‐quality PES membranes.^56^, ^57^ Because MBSA was found to be able to dissolve PES, it was tested for its ability to fabricate a PES membrane. The varying affinities of MBSA/PES casting solutions for solvents cause changes in morphology, leading to different performances of the produced membranes.

The membrane production process involves applying a degassed 10 wt % PES casting solution onto a glass plate. The glass plate is then submerged in a miscible non‐solvent to quickly remove the solvent, leaving a porous membrane. Traditionally, a dipolar aprotic solvent such as NMP is used as the solvent, which is removed by water as the non‐solvent. Because MBSA is immiscible with water and miscible with non‐polar solvents, a reversed approach was adapted for this work. Two non‐polar non‐solvents were chosen for this study, hexane and 2,2,5,5‐tetramethyloxolane (TMO),^58^ because both are miscible with MBSA. Water was also included in the study for comparison.

**Figure 2:**
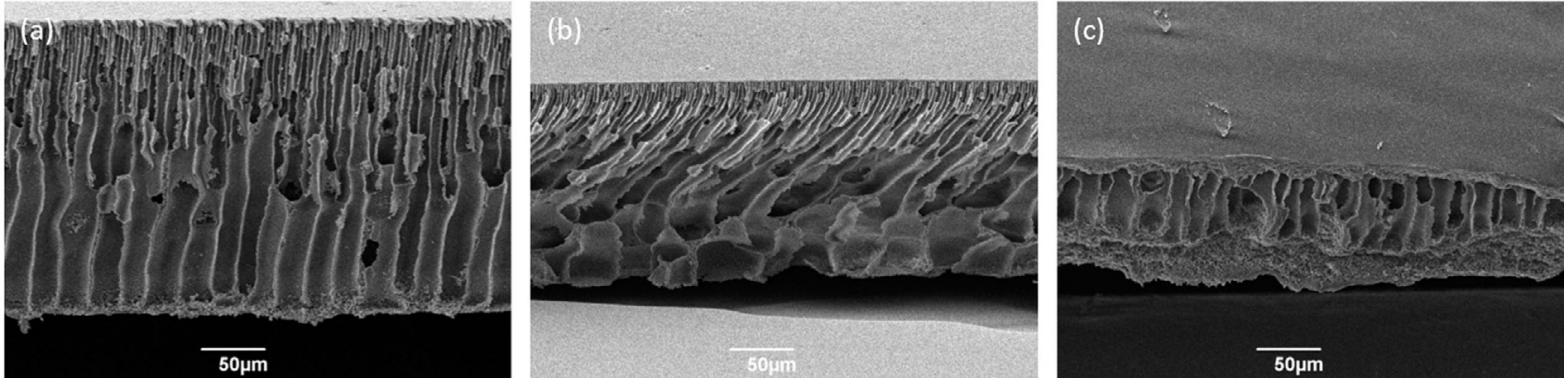
SEM images of cross‐sections of the membranes casted by using (a) water, (b) TMO, and (c) hexane as non‐solvent.

Demixing the PES/MBSA cast in hexane as the non‐solvent resulted in partial dissolution of the polymer (Figure 2 c). As a result, the morphology of the membrane was negatively affected, with dense regions at the surfaces. In addition, significant losses to the bulk solution of non‐solvent were also observed. Interestingly, a greener alternative to hexane, TMO, performed far better (Figure 2 b). It did not dissolve the polymer and allowed demixing of the mutually soluble MBSA, generating a finger‐like porous structure with large macro‐voids at the bottom. Using water as the non‐solvent generated a similar morphology to when TMO was used, but with slightly smaller macro‐voids at the bottom surface (Figure 2 a). Both morphologies are consistent with those previously reported in the literature.^46^, ^56^, ^57^ The performance of water as the non‐solvent was surprising because MBSA and water are immiscible. However, upon closer inspection, it was observed that water is partially soluble in MBSA (Figure S2 in the Supporting Information). Because the non‐solvent is in a large excess, effective demixing of the MBSA by water was achieved in this system.

The porosities of the PES/MBSA membranes produced by using TMO and water as non‐solvents were comparable to those previous reported in the literature^56^ and provide a fully green solvent system for their production.

#### MOF synthesis

MOFs are porous materials that have been demonstrated to be useful for many applications, from catalysis^59^ and gas absorption^60^ to electronics^61^ and sensors.^10^ As such, they can potentially be a vital cog in the green chemistry wheel. To be considered fully “green”, they must first be synthesized in a green way. Many MOFs are simply made by mixing the components together in a suitable solvent, so the solvent properties are the predominant factor in the greenness of the synthesis.^42^

Recently, the green dipolar aprotic solvent Cyrene has been demonstrated to be a suitable solvent to replace DMF for the synthesis of a selection of MOFs.^42^ Therefore, MOF synthesis could be an example of a promising application for the new succindiamide solvents. Two MOFs were chosen as probes, HKUST‐1 and ZIF‐8, because comparable data was already available for them.^42^ Their synthesis by using the succindiamides as the solvent in comparison to DMF was investigated.

Microwave heating was used in the preparation of the MOFs as an alternative to conventional heating. This shortened the MOF preparation time from 18 and 10 h for HKUST‐1 and ZIF‐8, respectively, to 20 min in the microwave.^42^ Although this already improved the greenness of the synthesis of the MOFs, more importantly, it demonstrated that the three new succindiamide solvents can absorb microwave energy, opening opportunities in other applications.

**Figure 3:**
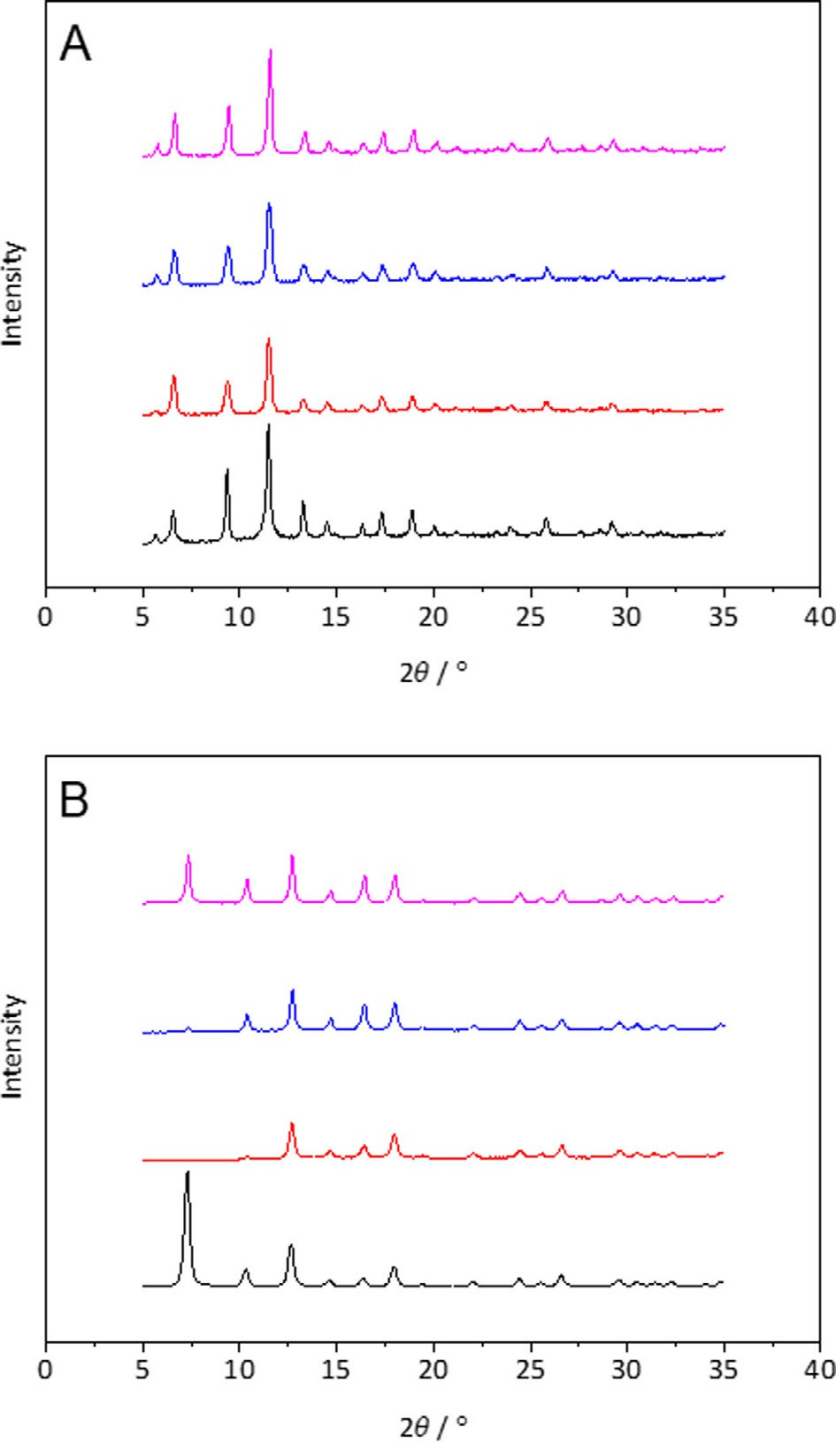
XRD spectra of HKUST‐1 (a) and ZIF‐8 (b) synthesized in DMF (black), MBSA (red), EBSA (blue), and TBSA (pink).

Figure 3 shows the powder XRD patterns for HKUST‐1 (a) and ZIF‐8 (b) MOFs produced in DMF, MBSA, EBSA, and TBSA. For HKUST‐1, it can be seen that the powder XRD pattern is almost identical in each solvent, indicating that the HKUST‐1 crystal structure is successfully synthesized in all new solvents. The peak widths in the crystals synthesized in EBSA were slightly broader, indicating a marginally smaller particle size. The intensity of the {2 2 2} reflection in MBSA (2 *θ=*11.4°) was similar to DMF, but lower in EBSA and TBSA. A lower intensity in {2 2 0} (2 *θ=*9.4°) but a greater intensity in {2 0 0} (2 *θ=*6.5°) was observed in all of the succindiamides compared with DMF, indicating a common preferential growth in the succindiamides that differed from DMF. The Brunauer–Emmett–Teller (BET) surface areas of HKUST‐1 produced in the different solvents are shown in Table 4 (isotherms can be seen in Figure S20 in the Supporting Information). EBSA generated the highest BET surface area (1116 m^2^ g^−1^) and was almost identical to that of DMF (1111 m^2^ g^−1^), whereas results for MBSA (981 m^2^ g^−1^) and TBSA (914 m^2^ g^−1^) were slightly lower.

**Table 4:** BET surface areas of the two MOFs synthesized in four solvents.

Solvent	*S*_BET_ HKUST‐1 [m^2^ g^−1^]	*S*_BET_ ZIF‐8 [m^2^ g^−1^]
MBSA	981	1137
EBSA	1116	667
TBSA	914	314
DMF	1111	1182

For ZIF‐8, only TBSA was successful in synthesizing the MOF with the same XRD pattern as in DMF (Figure 3 b). The {1 1 0} (2 *θ=*7.3°) peak was weak in EBSA and absent in MBSA, whereas the {2 0 0} (2 *θ=*10.3°) reflection was also weak in MBSA. The remaining pattern at higher 2 *θ* values closely resembled those in DMF. The porosity of the MOFs followed an opposite trend with MBSA (1137 m^2^ g^−1^) producing a comparable BET surface area to DMF (1182 m^2^ g^−1^), whereas EBSA (667 m^2^ g^−1^) and TBSA (314 m^2^ g^−1^) produced lower BET surface areas.

Thermogravimetric analysis (TGA) traces of the four ZIF‐8 samples suggest that the reason for the lower BET surface areas of ZIF‐8 synthesized in TBSA and EBSA is that residual solvent may have been trapped in the pores (Figure S21 in the Supporting Information). Mass losses at approximately 400 °C in the EBSA sample and approximately 500 °C for the TBSA sample suggest the evaporation of trapped solvent. These mass losses were not observed in the DMF or MBSA samples.

#### Heck reaction

**Scheme 2:**
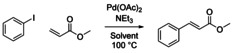
Heck reaction between iodobenzene and methyl acrylate.

**Figure 4:**
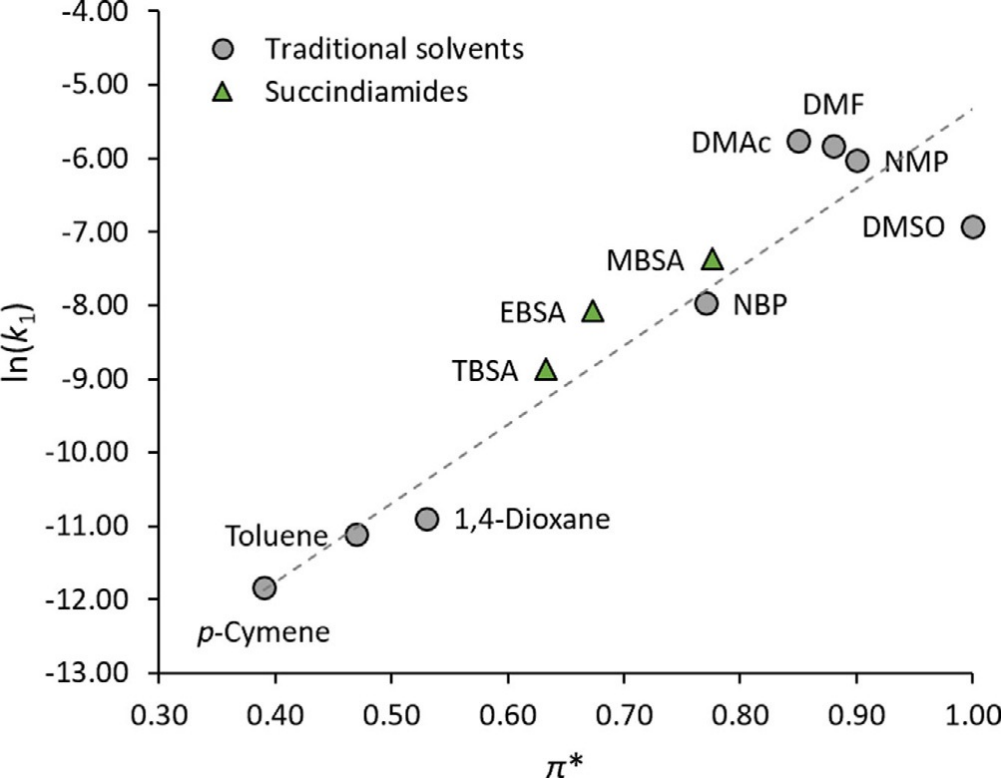
LSER showing the reaction rates of the Heck reaction in a range of solvents.

The Heck reaction is a pharmaceutically relevant reaction that is also dependent on solvent polarity, being promoted in polar solvents.^4^, ^24^ As such, succindiamides are applied as solvents for this reaction to evaluate their suitability for Heck, or indeed C−C‐coupling reactions in general. A model Heck reaction between methyl acrylate and iodobenzene was performed in different solvents (Scheme 2). Using DMSO as a solvent, the reaction order was confirmed to be first‐order with respect to methyl acrylate.^62^ A linear solvation‐energy relationship (LSER) of the natural log of the first‐order rate constant [ln(*k*_1_)] versus π* of a range of solvents can be seen in Figure 4 and illustrates the rate dependence on solvent polarity of the model Heck reaction.

MBSA was particularly effective for this reaction, performing comparably to DMSO and better than NBP. TBSA and EBSA fitted the trend and performed according to their polarity. Interestingly, during the reaction it was observed that the triethylammonium iodide salt formed during the coupling precipitated out of solution in the three succindiamides in the course of the reaction. In contrast, the traditional dipolar aprotic solvents kept the ammonium salt in solution throughout the reaction. This is potentially very useful because it makes product isolation easier compared with traditional dipolar aprotic solvents. Again, this highlights the lack of ionic character and hydrogen‐bonding ability in the succindiamides, an unusual property that may be beneficial in many future chemical processes.

### Toxicity testing

To examine the effect of the *N*‐butylamide group in comparison to the *N*‐methylamide group in terms of their toxicities, an integrated approach using both in silico and in vitro assessments was performed. Details about the materials and methods can be found in the Supporting Information.

The in silico approach consisted of gathering any available adequate experimental toxicity data for CMRS endpoints, performing quantitative structure–activity relationship (QSAR) model‐based predictions by using Vega Hub,^63^ Danish QSAR database,^64^ and Toxtree tools, and exploring read‐across from similar structures with adequate experimental toxicity data or available QSAR predictions (in the Danish QSAR database).

The in vitro approach utilized the CALUX® battery of 18 in vitro reporter gene assays, covering a broad range of toxicological endpoints, providing information on the propensity of a test compound to trigger certain molecular events, which could result in adverse health effects. This panel has been used successfully in several large screening programs, such as the EU Framework program (FP) ReProTect and ChemScreen projects, which were both specifically directed at the detection of reproductive toxicity.^65^, ^66^, ^67^, ^68^, ^69^

Complementarity was based on the notion that the in silico models are using structural alerts of chemicals to predict biological behavior, whereas the in vitro methods use biological pathways to assess chemical behavior in a more unbiased manner.

#### In silico toxicity analysis

If experimental data of sufficient quality was available for the candidate compound, these were taken as decisive for the health endpoint of that compound, that is, indicating the presence or absence of specific hazardous properties. These data therefore overruled the in silico model predictions and prevented any further read‐across explorations. Such was the case for NBP: experimental data for M, R, and S were available and adequate, and all indicated that NBP was negative for these endpoints. For NBP, therefore, in silico predictions were only performed for C, which was also found to be negative. This is illustrated in Table 5 by “−” for C, M, R, and S for NBP. Table 5 also shows NMP as positive for R, and negative for C, M, and S.

**Table 5:** CMR and S assessments for NMP and its candidate substitute compounds.

Compound	C	M	R	S
NMP	− (exp)	− (exp)	+ (exp)	− (exp)
NBP	−	− (exp)	− (exp)	− (exp)
MBSA	−	?	−	−
EBSA	−	?	−	−
TBSA	−	?	−	−

“−”: absence of property; “+”: presence of property; “?”: no prediction possible; “exp”: conclusion based on reliable experimental data.

No experimental data were available for any of the butylsuccindiamides in this work and, thus, QSAR model predictions were generated for all four toxicological endpoints. Because predictions in the Danish QSAR database for these specific butylsuccindiamides were not available, predictions that were available for the structural analogues tetramethyl‐ and tetraethylsuccindiamides (with CAS 7334‐51‐2 and 22692‐57‐5, respectively) were used instead: both were predicted to be negative for C and R, whereas predictions for M (chromosomal aberrations) and S were out of domain.

Vega Hub predictions for the butylsuccindiamides were out of domain for C, negative for M (i.e., for bacterial mutagenesis), negative for R, and not trustworthy for S. The overall conclusion for M, combining predictions from the Danish QSAR database and Vega Hub, was inconclusive, reflected by “?” in Table 5. Because the succindiamide structure is not an alert for S,^70^ this endpoint is predicted negative as well, indicated by “−”. Thus, Table 5 shows that NBP, the candidate that is structurally closest to NMP, received a negative score for all CMR and S endpoints, based on reliable experimental data (“exp”) for M, R, and S, and on an in silico prediction for C. The CMRS assessment for the other three candidate compounds MBSA, EBSA, and TBSA, structurally less close to NMP but structurally closely related among themselves, also showed negative predictions for all four endpoints

#### In vitro reporter gene assay analysis

NMP, NBP, MBSA, EBSA, and TBSA were analyzed on a panel of 18 reporter gene assays, covering different toxicological endpoints (Table 6). All compounds showed cytotoxicity in the millimolar range; for the succindiamides, the lowest effect concentration (LEC), which reflects the compound's potency, increased with increasing chain length from 5.0 to 0.4 mm. The lowest cytotoxicity was observed for NMP (40 mm). However, because the succindiamides are poorly soluble once transferred to the aqueous cell culture medium, this relatively low observed cytotoxicity could be an underestimation: if only 10 % of the succindiamides was in solution, the concentration able to activate the cellular assays was in reality even 10 times lower than the reported values in Table 6, corresponding to a 10 times higher potency.

**Table 6:** CALUX assay results presented as LECs in Log *M*.

Test	NMP	NBP	MBSA	EBSA	TBSA
cytotoxicity	−1.4	−2.1	−2.3	−3.1	−3.4
PXR	–	−3.2	−3.9	−5.0	−6.1
ERα	–	–	–	–	–
AR‐anti	–	–	–	–	–
PR‐anti	–	–	–	–	–
GR‐anti	–	–	–	–	–
TRβ	–	–	–	–	–
TRβ‐anti	–	–	–	–	–
AhR	−2.0	–	–	–	–
PPARα	–	–	–	–	–
PPARδ	−2.2	−2.3	–	–	–
PPARγ	–	–	–	–	–
TCF	−2.1	–	–	–	–
AP1	−1.5	–	–	–	–
ESRE	–	–	−2.4	–	–
Nrf2	–	–	−2.9	–	–
p21	−2.0	–	–	–	–
p53	–	–	–	–	–

(–)=no effect observed up to the highest test concentration.

The CALUX assays listed in Table 6 detect the ability of a test compound to modulate activation of a certain nuclear receptor (PXR through PPARγ), or a cell signaling pathway (TCF through p53). Because these early molecular events are often involved in multiple adverse outcome pathways, it is not always straightforward to link each assay to a specific toxicological endpoint. Nonetheless, when focusing on the CMR endpoints that are prioritized in REACH legislation, several molecular targets have been shown to be relevant for these endpoints.

The PXR CALUX is a xenobiotic sensor; the fact that compounds activate this assay indicates that they are recognized as non‐endogenous to the cells. PXR activation leads to the induction of metabolic enzymes, resulting in enhanced metabolism of a wide range of compounds. Its activation has been correlated with a protective effect against reproductive toxicity.^71^ The CALUX results show that NBP activated PXR, whereas NMP was negative. This is in line with the fact that NMP is a known reprotoxicant, whereas NBP has been tested negative in terms of reproductive toxicity.^28^ The three succindiamides were all able to activate PXR, which may indicate that these chemicals are less likely to induce reproductive toxicity.

No activity was observed on the endocrine assays, which measure activation of nuclear hormone receptors (estrogen, androgen, progesterone, glucocorticoid, and thyroid) and are often involved in reproductive toxicity.^71^ Other receptors that may be relevant in reproductive toxicity, like PPARs^71^ and AhR, were activated by NMP only (AhR) or NMP and NBP (PPARδ).

Six of the CALUX assays (TCF through p53) detect activation of several cellular signaling pathways, which are indicative of general stress and acute toxicity, but also a range of more specific types of toxicity, including reproductive toxicity. NMP activates three of these assays, which can be linked to reproductive toxicity [Wnt signaling (TCF)],^72^ cell cycle control (AP‐1), or DNA damage response (p21).^73^ NBP did not activate any of these assays. Of the succindiamides, only MBSA showed activity on two of the cellular signaling pathway assays: ESRE (unfolded protein response) and Nrf2 (oxidative stress).

Overall, the in vitro analysis showed that the succindiamides activate fewer assays than NMP, but generally at much lower concentrations, suggesting a higher potency. For NMP, the LECs are 3–40 mm; for MBSA, 0.1–5.0 mm; for EBSA, 0.01–0.80 mm; and for TBSA, even 0.001–0.400 mm. The assays activated by the succindiamides do not show clear indications for reproductive toxicity. On the contrary: PXR activation, observed for all three succindiamides, has been shown to be inversely correlated with reproductive toxicity;^71^ as such, the PXR activation at micromolar concentrations by EBSA and TBSA could be a favorable characteristic.

When comparing the succindiamides to each other, two opposing trends are observed. The number of active assays decreases with increasing chain length [MBSA (4)>EBSA (2)=TBSA (2)], whereas the potency increases with increasing chain length (LOECs MBSA 0.1–5.0 mm; EBSA 0.01–0.80 mm; TBSA 0.001–0.400 mm).

## Conclusions

Amide solvents have received negative publicity in recent years owing to their toxicity, with *N*,*N*‐dimethylformamide (DMF), *N*‐methyl‐2‐pyrrolidone (NMP), and *N*,*N*‐dimethylacetamide (DMAc) being classed as substances of very high concern (SVHC) by REACH (Registration, Evaluation, Authorization, and Restriction of Chemicals) owing to their reprotoxicity. The target of this work was to find non‐reprotoxic but highly dipolar biobased or bioderivable molecules to replace traditional dipolar aprotic solvents. A set of molecules with *N*‐butylamide functionality was identified as being a likely route to this objective owing to the presence of two amide groups (high dipolarity) with *N*‐butyl alkyl chains (low reprotoxicity). Three succindiamide solvents were synthesized, *N*,*N*,*N*′,*N*′‐tetrabutylsuccindiamide (TBSA), *N*,*N*′‐diethyl‐*N*,*N*′‐dibutylsuccindiamide (EBSA), and *N*,*N*′‐dimethyl‐*N*,*N*′‐dibutylsuccindiamide (MBSA). All are produced from the biobased platform molecule succinic acid and alkylbutylamines. To produce 100 % biobased solvents, the alkylbutylamines can be synthesized from bio‐butanol and a biobased version of methanol or ethanol.

The succindiamides displayed some unusual properties. Interestingly, all three were immiscible with water but miscible with the non‐polar hexane, which is highly uncommon for a dipolar aprotic solvent. The solvents were trialed in the dissolution of industrially relevant polymers [polyvinylidene fluoride (PVDF), polyethersulfone (PES), and polyamide imides (PAIs)], which currently rely on NMP, DMF, or DMAc in a number of applications. All three were shown to dissolve high molecular weight PVDF and PAI at elevated temperatures, whereas MBSA can also dissolve PES for the fabrication of an industrially relevant membrane. Future work should look at utilizing these solvents in applications such as Li battery binders, wire enameling, and as cosolvents in membrane formation.

Additionally, a model Heck reaction and two metal–organic framework syntheses were performed, in which comparable performances to traditional solvents were observed when using the succindiamides. An effect of the water immiscibility was observed in the Heck reaction: the ammonium salt produced as a byproduct precipitated out of solution, benefitting product isolation.

The toxicity of the succindiamides was assessed by using an integrated approach consisting of in silico analysis based on available experimental information, prediction model outcomes, and read‐across data, combined with a panel of in vitro reporter gene assays covering a broad range of toxicological endpoints. Assessment of the in silico predictions and data resulted in none of the succindiamides being likely to exhibit carcinogenicity (C), mutagenicity (M), reproduction toxicity (R), or skin sensitization (S) properties. In addition, the in vitro tests suggested no alarming indications of toxicity, and their activation profile compares favorably to that of NMP, but the analysis should be regarded with some caution because of the poor water miscibility of the compounds.

Overall, despite not possessing as high dipolarity as targeted from the outset of this work, TBSA, EBSA, and MBSA performed well in several applications including some common synthetic reactions and solubility tests. They can claim to be green in several criteria, being produced catalytically from biomass, and compare favorably to NMP based on in silico and in vitro toxicity testing, which showed no significant indications of CMRS activity.

Finally, the observed unusual water immiscibility makes them interesting candidates for further research in a variety of applications.

## Conflict of interest

*The authors declare no conflict of interest*.

## Supporting information

As a service to our authors and readers, this journal provides supporting information supplied by the authors. Such materials are peer reviewed and may be re‐organized for online delivery, but are not copy‐edited or typeset. Technical support issues arising from supporting information (other than missing files) should be addressed to the authors.

Supplementary
