# Supplementary material for: A Family of Water‐Immiscible, Dipolar Aprotic, Diamide Solvents from Succinic Acid
Source: ChemSusChem. 2020 May 11;13(12):3212–21. doi: 10.1002/cssc.202000462 (PMC7318222; doi:10.1002/cssc.202000462)
Supplement: Supplementary file 1 — Supplementary [file CSSC-13-3212-s001.pdf]

ChemSusChem

Supporting Information

A Family of Water-Immiscible, Dipolar Aprotic, Diamide Solvents from Succinic Acid

Fergal P. Byrne,*[a] Clara M. Nussbaumer,[a] Elise J. Savin,[a] Roxana A. Milescu,[a] Con R. McElroy,[a] James H. Clark,[a] Barbara M. A. van Vugt-Lussenburg,[b] Bart van der Burg,[b] Marie Y. Meima,[c] Harrie E. Buist,[c] E. Dinant Kroese,[c] Andrew J. Hunt,[d] and Thomas J. Farmer[a]

Materials and methods

Materials

Succinyl chloride (2 M soln. in dichloromethane), *N*-methylbutylamine 90%, *N*-ethylbutylamine 95%, Dibutylamine 99% were purchased from TCI chemicals. K60 silica was purchased from Merck chemicals. Succinic acid ≥99.0%, *N*-methylpyrrolidinone 99.0%, 1-bromooctane ≥97.0%, 1-methylimidazole 99.0%, iodobenzene 99.9%, methylacrylate 99.9%, palladium(II) acetate ≥99.9% trace metals basis, triethylamine ≥99.0%, trimesic acid 95%, copper(II) nitrate trihydrate ≥99.0%, 2-methylimidazole 99.0%, zinc(II) nitrate hexahydrate ≥99.0%, *para*-cymene 99.0%, 1,4-dioxane 99.8% (anhydrous), 4-nitroaniline ≥99.0%, 1-octanol ≥99.0%, diglyme ≥99.0%, DMSO-d_6 (99.8% D) and chloroform-d (99.8% D) were purchased from Sigma-Aldrich. Toluene ≥99.0%, hexane ≥99.5%, DCM ≥99.5%, chloroform ≥99.5%, DMSO ≥99.0% were purchased from VWR. *N*,*N*-diethyl-4-nitroaniline ≥98.0% was purchased from Alfa Aeser. Cyrene was supplied by Circa group. *N*-butylpyrrolidinone was supplied by Eastman chemicals. *N*,*N*-dimethylformamide 99.9% and *N*,*N*-dimethylacetamide 99.9% were purchased from Fischer. The flakes of Ultrason® E3020 P Polyarylethersulfone of 55,000 Da were obtained from INGE.BASF, Germany. The CALUX cell lines have been described previously.[1] They are based on human U2-OS cells (ATCC, HTB-96) stably transfected with an expression construct for the full-length human receptor under investigation, and a reporter construct consisting of multimerized responsive elements for the cognate receptor coupled to a minimal promoter element (TATA) and a luciferase gene. Cells were maintained as described previously,[2] using cell culture media and supplements from Fisher Scientific as described in the experimental procedures section.

Equipment

Gas chromatograph-mass spectrometry (GC-MS) was carried out on a Perkin Elmer Clarus 500 GC with a Clarus 560 S quadrupole mass spectrometer. The equipment was fitted with a ZB5-HT capillary column (30 m x 250 μm x 0.25 μm nominal, max temperature 430 °C). Helium was used as the carrier gas with flow rate of 1.0 mL/min, and a split ratio of 10:1. The injector temperature was 330 °C. The initial oven temperature was 50 °C which was held for 4 minutes. The temperature increased at a rate of 10 °C/min as far as 300 °C at which point it was held for 10 minutes. The Clarus 500 quadrupole mass spectrum was conducted in electron ionisation (EI) mode at 70 eV with the source temperature and the quadrupole both at 300 °C. The *m/z* mass scan was in the range of 40 to

640 *m/z*. The data was collected by the PerkinElmer enhanced TurboMass (Ver. 5.4.2) chemical software. Each GC-MS sample consisted of ~10 mg product mixture in 1.5 mL methanol or hexane as GC-MS solvent.

^{1}H NMR and ^{13}C NMR spectra were measured on a JEOL JNM-ECS 400 MHz spectrometer. 16 scans were used for ^{1}H NMR analysis, and 256 scans were used for ^{13}C NMR analysis. The NMR data were processed and analysed by ACD/NMR Processor Academic Edition software (Ver. 12.01).

The UV vis. spectra were measured on a JENWAY, 6705 UV/Vis. spectrophotometer in quartz cuvettes at 25 °C.

An Agilent 6890N gas chromatograph with a flame ionisation detector (GC-FID), fitted with a ZB-5HT capillary column (30 m x 250 μm x 0.25 μm nominal, max temperature 400 °C) was used in this work. Helium was used as the carrier gas at a flow rate of 2.0 mL/min. The split ratio was 50:1. The initial oven temperature was 40 °C which was held for 5 minutes at which point it was increased at a rate of 10 °C/min to 250 °C. Injection temperature was 250 °C and the detector temperature was 250 °C.

DSC was carried out on a TA Instruments DSC Q2000 machine. Samples were placed in a Tzero alodined pan and hermetically sealed alodined lid under a nitrogen flow of 50 mL min^{-1}. The initial temperature was 30 °C and was ramped down to -90 °C and back to 30 °C at 5 °C min^{-1} both ways. This ramp rate was repeated for a second cycle.

A CEM Discover microwave was used in this work. Experiments were carried out in 15 mL and 35 mL vials.

Porosimetry was carried out on a Micromeritics TriStar Surface Area and Porosity Analyser. A measured amount of dry (ca. 100 mg), powdered sample was weighed into a porosimetry tube. The samples were degassed at 110 °C for 6 h and the combined mass of the glassware and sample was re-measured. The data was processed using TriStar software, where specific surface areas were calculated using the Brunauer-Emmett-Teller (BET) equation, and desorption pore volume and average pore size were calculated using Barrett-Joyner-Halenda (BJH) equations.[3]

Thermogravimetric analysis was carried out on a Stanton Redcroft Simultaneous Thermal Analyzer, STA 625 at scan rates of 10 °C min^{-1}, with ~10 mg sample under flowing N_2 at 60 mL min^{-1}.

XRD analysis was performed on a Bruker-AXS D8 Advance Diffractometer equipped with a Cu source producing a monochromatic K-α radiation at wavelength of 1.54184 Å and a PSD Lynx eye detector. Samples were ground to a fine powder prior to analysis. Samples were run at a rate of 2.0° min^{-1} over a 2θ range of 5–38° (cellulose does not present any diffraction pattern after this angle)[4] in a locked coupled theta–2θ scan mode. Generator voltage and current 77 were set to 40 kV and 40 mA respectively. Data processing included background subtraction and trace smoothing. The crystalline index (CrI) of MFC samples was calculated according to Segal's equation (Equation S1).[5]

Equation S1. $$CrI_{\%} = \frac{I_{200} - I_{am}}{I_{200}} \times 100$$

Where I_{200} = intensity of the (200) peak (at 2θ = 22° ±0.5), I_{am} = intensity of amorphous contributions (at 2θ = 18.0° ±0.5).

The equipment used for toxicity testing is described in the procedure for the toxicity testing (section "In vitro reporter gene assays").

Experimental procedures

Synthesis of succindiamide solvents from succinyl chloride

The corresponding *N*-alkylbutylamine (1.28 mol) and dichloromethane (250 mL) were added to a 1 L three-necked flask fitted with a dropping funnel and stirred with a magnetic stirring bar. A solution of succinyl chloride (35.17 mL, 0.32 mol) in dichloromethane (200 mL) was added dropwise at 0 °C under a flow of nitrogen. The system was purged with nitrogen using a balloon and the released HCl gas was scrubbed using a 1 M NaOH solution in a Dreschel bottle. The reaction mixture was stirred overnight, after which it was washed with aqueous hydrochloric acid solution (1M, 3 × 250 mL), aqueous sodium hydroxide solution (1M, 2 × 125 mL) and brine (2 × 200 mL). The organic phase was dried with magnesium sulfate and the solvent was removed by rotary evaporation. The product MBSA was purified via Kugelrohr distillation. The first fraction was obtained at 150 °C and 0.8 mbar pressure. The product-fraction was separated from the residue at 220 °C and 0.8 mbar pressure. The obtained yellowish-green product was analysed via NMR spectroscopy (JEOL JNM-ECS 400), GC-FID (Agilent 6890N), GC-MS (PerkinElmer Clarus 560 S) and IR spectroscopy (PerkinElmer Spectrum 400).

[*N*,*N*'-Dimethyl-*N*,*N*'-dibutylsuccindiamide (MBSA)] ^{1}H NMR (400 MHz; $CDCl_3$): δ 0.83 (6H, m, 2 × CH_3), 1.21 (4H, m, 2 × CH_2), 1.43 (4H, m, 2 × CH_2), 2.57 (4H, m, 2 × CH_2), 2.82 (3H, s, CH_3), 2.93 (3H, s, CH_3), 3.24 (4H, m, 2 × CH_2). ^{13}C NMR (400 MHz, $CDCl_3$): δ 13.39, 19.51, 27.52, 28.09, 28.95, 29.96, 32.98, 34.67, 47.13, 49.14, 171.28. GC-MS (EI): *m/z* 256 (M+•). IR: 2957, 2929, 2872, 1635, 1457, 1398, 1295, 1266, 1214, 1135, 1085 cm^{-1}.

[*N*,*N*'-Diethyl-*N*,*N*'-dibutylsuccindiamide (EBSA)] ^{1}H NMR (400 MHz; $CDCl_3$): δ 0.88 (6H, m, 2 × CH_3), 1.05 (3H, m, CH_3), 1.14 (3H, m, CH_3), 1.27 (4H, m, 2 × CH_2), 1.49 (4H, m, 2 × CH_2), 2.63 (3H, t, 2 × CH_2), 3.24 (4H, m, 2 × CH_2), 3.32 (4H, m, 2 × CH_2). ^{13}C NMR (400 MHz, $CDCl_3$): δ 12.62, 13.49, 13.75, 27.88, 29.68, 30.78, 40.40, 41.87, 45.01, 46.96, 171.05. GC-MS (EI): *m/z* 284 (M+•). IR: 2958, 2931, 2873, 1634, 1455, 1425, 1375, 1313, 1277, 1247, 1209, 1134, 1113, 1099, 1076 cm^{-1}.

[*N*,*N*,*N*',*N*'-Tetrabutylsuccindiamide (TBSA)] ^{1}H NMR (400 MHz; $CDCl_3$): δ 0.89 (12H, m, 4 × CH_3), 1.27 (8H, m, 4 × CH_2), 1.50 (8H, m, 4 × CH_2), 2.65 (4H, s, 2 × CH_2), 3.25 (8H, m, 4 × CH_2). ^{13}C NMR (400 MHz, $CDCl_3$): δ 13.59, 19.94, 28.07, 29.67, 30.80, 45.64, 47.43, 171.35. GC-MS (EI): *m/z* 340 (M+•). IR: 2957, 2929, 2872, 1637, 1455, 1424, 1374, 1286, 1255, 1218, 1199, 1136, 1113 cm^{-1}.

Synthesis of succindiamides from succinic acid

The corresponding *N*-alkylbutylamine (0.225 mol, 15 eq.), succinic acid (1.77 g, 0.015 mol) and K60-700 silica catalyst (1.06 g, 60 w% of succinic acid) (prepared by calcining K60 silica at 700 °C for 4 hours) were added to a 100 mL round-bottomed flask. The reaction mixture was stirred under reflux for 28 h. Upon completion of the reaction, the viscous mixture was distilled by Kugelrohr short-path distillation at 0.8 mbar and the yellow product was analysed by NMR spectroscopy. The catalyst was recovered by calcination at 700 °C for 5 h.

Synthesis of N,N'-Dimethyl-N,N'-dibutylsuccindiamide in a closed vessel

N-Methylbutylamine (3.92 mL, 45 mmol, 15 eq.), succinic acid (0.354 g, 3 mmol) and K60-700 silica catalyst (212 mg, 60 wt% of succinic acid) (prepared by calcining K60 silica at 700 °C for 4 hours) were added to a 20 mL Hastelloy pressure-vessel. The reaction mixture was stirred under reflux for 28 h. Upon completion of the reaction, pressure was released and the viscous mixture was distilled by Kugelrohr short-path distillation at 0.8 mbar and the yellow product was analysed by NMR spectroscopy.

Determination of Kamlet-Taft parameters

The Kamlet-Taft parameters were measured by dissolving *N*,*N*-diethyl-4-nitroaniline (NN) and 4-nitroaniline (NA) dyes in the test solvent (TS) and scanning on the UV vis. spectrophotometer to determine $\nu_{\max}$ (NA) and $\nu_{\max}$ (NA). π^* and β were then calculated using Equation S2 and S3 respectively.

Equation S2. $$\pi^* = \frac{\nu_{\max(NN)}[TS] - \nu_{\max(NN)}[cyclohexane]}{\nu_{\max(NN)}[DMSO] - \nu_{\max(NN)}[cyclohexane]}$$

Equation S3. $$\beta = 0.74\,\frac{\nu_{\text{Calculated}}[TS] - \nu_{Observed}[TS]}{\nu_{\text{Calculated}}[DMSO] - \nu_{Observed}[DMSO]}$$

The $\nu_{\text{Calculated}}$ represents the $\nu_{\max}$ predicted by a baseline of non-hydrogen-bonding solvents. Deviations from this baseline are proportional to β. Equation S4 shows baseline used in this work to find β was that which was determined by Sherwood.[6] R^2 is shown in Equation S5.

Equation S4. $$y = 1.0025x + 3.4426$$

Equation S5. $$R^2 = 0.9945$$

HSPiP software predictions

HSPiP (4th Edition 4.1.04) is a computer modelling software which can predict the Hansen solubility parameters (HSPs) of an inputted molecule. It was used to predict the HSPs of the three succindiamide solvents in this work.

Density measurements

The density of each of the succindiamides was determined by weighing 1 mL of the solvent on a Kern 120 g balance. Toluene and water were used as reference samples.

Determination of octanol/water partition coefficient (Log $P_{(o/w)}$)

Determination of the log $P_{(o/w)}$ was done by the shake flask method. 1.5 mL each of octanol and water were mixed in a 4 mL vial. 30 µL of the test solute and 10 µL of an internal standard (diglyme) were added and the mixture was shaken for 30 seconds and allowed to stand overnight. Samples (1 mL) were taken from both the aqueous and organic layers and added to 1.5 mL GC vials for GC analysis. Log $P_{(o/w)}$ was obtained using Equation S5.

Equation S5. $$Log\ P_{(o/w)} = k_{IS}\frac{Area(sample)_o}{Area(sample)_w}\frac{Area(IS)_w}{Area(IS)_o}$$

Membrane fabrication

The casting solution was prepared by dissolving 10 wt% of PES pellets (relative to the mass of solvent) in MBSA at 100 °C for 6h. The casting solution was degassed, then placed on a glass plate using a manual casting knife with a thickness of 150 μm. The plate containing the casted polymer was immersed in a bath of non-solvent in a non-solvent phase inversion technique (NIPS), causing the PES membrane to precipitate.

Determination of hexane and water miscibility

Miscibility tests were carried out by mixing 1 mL of each of the test solvents in a 2.5 mL vial, shaking vigorously for 10 seconds and allowing to stand. Mixtures which formed one phase were classed as miscible and those which formed two phases were classed as immiscible.

Heck reaction (Synthesis of methyl cinnamate)

Iodobenzene (1.67 mL, 15 mmol), methyl acrylate (1.63 mL, 18 mmol), triethylamine (2.51 mL, 18 mmol) and the chosen solvent (6 mL) were added to a 25 mL round-bottomed flask and pre-heated to 100 °C and stirred with a magnetic stirring bead at 500 rpm. Palladium acetate (10 mg, 3 mol%) was added to the flask to begin the reaction. Aliquots were taken after 30-60 minutes to determine conversion by NMR spectroscopy.

Metal-organic framework (MOF) synthesis

Metal-organic frameworks (MOF) were prepared similarly to the method of Zhang et al.[7] Microwave heating was used instead of conventional heating which allowed a reaction time of 20 minutes instead of 24 hours. Detailed procedures are described below.

HKUST-1

Trimesic acid (100 mg, 0.48 mmol), copper(II) nitrate trihydrate (172 mg, 0.70 mmol) and the test solvent mixture were added to a 35 mL microwave vial. For the succindiamides, the solvent mixture was 8 mL ethanol and 8 mL succindiamide; for DMF, the solvent mixture was 8 mL DMF, 8 mL ethanol and 8 mL water. The mixture was heated to 80 °C in a CEM Discover microwave for 20 minutes. The resulting solid was solvent exchanged daily with DMF (x1) and ethanol (x5). The solid was then dried in the oven at 110 °C overnight to yield a purple powder.

ZIF-8

2-Methylimidazole (320 mg, 3.87 mmol), zinc(II) nitrate hexahydrate and 24 mL solvent were added to a 35 mL microwave vial. Once dissolved, triethylamine (0.576 mL, 7.74 mmol, 2 eq.) were added and the mixture was heated to 100 °C. The resulting solid was solvent exchanged daily with DMF (x1) and ethanol (x5). The solid was then dried in the oven at 110 °C overnight to yield a white powder.

In silico predictions

Prediction tools applied

VEGA[8] provides access to a series of QSAR models on different properties. VEGA uses both statistical models and rule-based methods, which is preferable to either only a statistical or only a rule based method. Predictions can be done by entering the SMILES code of the desired compound. VEGA provides a report with the compound predictions of 'toxicant' or 'non toxicant' and a reliability score indicating the compound being (possibly) out of applicability domain. Moreover, VEGA reports provide the structures of the compounds to which the unknown chemical is compared to.

The Danish (Q)SAR Database (DTU)[9] includes predictions of toxicity produced by more than 200 (Q)SARs from open-access and commercial platforms for more than 600,000 chemical substances. The Danish (Q)SAR Database used three QSAR software models for predicting toxicity of chemicals, i.e. CASE Ultra, Leadscope Predictive Data Miner (Leadscope) and SciQSAR. Based on the predictions of these systems, a battery prediction is (often) made which combines these systems to obtain more reliable predictions (Manual for the Danish (Q)SAR Database, freely downloadable at http://qsar.food.dtu.dk/). Thus, this database contains predictions for a large set of compounds and cannot be used if a compound is not part of this set. The outcome for the model predictions are negative, positive or out of domain.

Toxtree[10] is an open source application which is able to estimate toxic hazard by applying a decision tree approach. The tool predicts toxic hazard based on the molecular structure of a compound on the basis of a rule-based system.[11] The tool does not provide information on whether a predicted chemical is inside or outside the applicability domain, which is a disadvantage. Therefore, it is highly relevant to calculate model performances. In the tool, the SMILES code of a compound can be entered. The tool then provides an outcome for a selected model. The outcome of a model prediction for a given compound within Toxtree provides two possibilities: positive (e.g. predicted toxic/structural alert) or negative (e.g. predicted nontoxic/no structural alert).

In vitro reporter gene assays

The automated CALUX assays were carried out similarly as described earlier,[12] with some adaptations. In brief, the assay was performed in CO_2 independent medium (Fisher Scientific, 11580536) supplemented with 5% DCC serum, 1x NEAA (Fisher Scientific, 11350912), 1x GlutaMAX supplement (Fisher Scientific, 11574466), 10 U/mL penicillin and 10 µg/mL streptomycin. A cell suspension in assay medium was made of 1×10^5cells/mL, and white 384-wells plates were seeded with 30 µl cell suspension/well. After 24 h, exposure medium was prepared. A dilution series in 0.5log unit increments of each test compound (in DMSO) was added to a 96-wells plate containing assay medium (2% v/v). Of this exposure mixture, 30 µl was added to the assay plates containing the CALUX cells, resulting in a final DMSO concentration of 1%. Additionally, DMSO blanks and a full dose response curve of the relevant reference compound were included on each plate. All samples were tested in triplicate. The preparation of the compound dilution series as well as the exposure of the cells were performed on a Hamilton StarLET liquid handling robot coupled to a Cytomat incubator. Directly after exposure, plates were sealed with non-breathing plate seals (Plate sealer, easy seal transparent; Greiner-Bio-One, 676001). After 24 h the exposure medium was removed using an EL406 washer-dispenser (BioTek) and 10 µl/well triton lysisbuffer was added by the EL406. Subsequently, the luciferase signal was measured in a luminometer (InfinitePro coupled to a Connect Stacker, both TECAN). In order to be able to detect receptor antagonism, several assays were also performed in antagonistic mode. The assay procedure was as described above, with the only exception that the EC50 concentration of the reference agonist was present during the exposure.

The luminometer data was analysed as follows; the average of the triplicate wells was determined, and the average blank (DMSO) value was subtracted. For receptor-based assays the maximum response elicited by the reference compound was set to 100% (full receptor- or pathway activation), and the other values were scaled accordingly. GraphPad Prism was used to fit a sigmoidal curve through the data (four parameters, variable slope). The PC10 concentration was reported as the lowest effect concentration (LEC), which is defined as the concentration where the response elicited by the test compound equals 10% of the maximum response of the reference compound. For antagonist- and cytotoxicity experiments, PC20 values were determined instead, which was defined as the concentration where the test compound causes a 20% decrease in the basal signal, which was set to 100%. For cell signalling pathway based assays that do not generate a sigmoidal dose-response curve, the signal induction above background (DMSO) was determined, and the concentration where the compound elicited a 1.5-fold increase above background was used as LEC.

Industrially relevant polymer dissolution study photographs

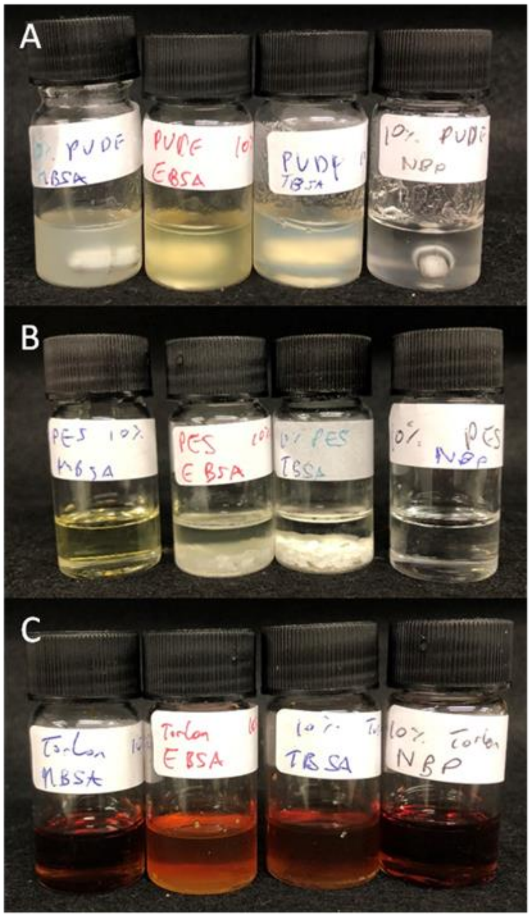

Figure S1. Polymer dissolution at 10 wt% of (A) PVDF (B) PES (C) PAI in (left to right) MBSA, EBSA, TBSA and NBP. Dissolution carried out at 80 °C with agitation for 1 hours, images taken at room temperature.

Succindiamide/water miscibility photographs

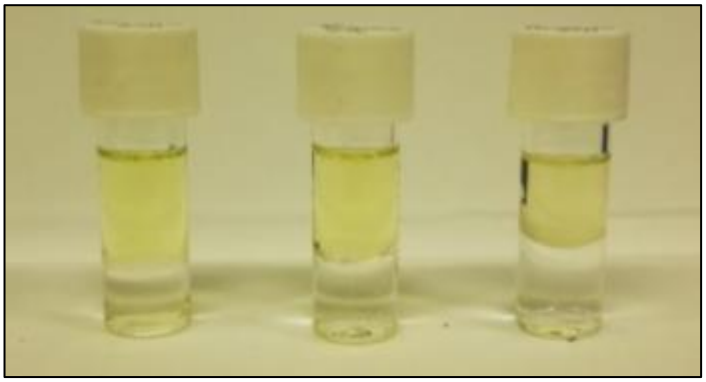

Figure S2. Miscibility of succindiamide solvents with water – MBSA (left), EBSA (centre) and TBSA (right).

NMR spectra

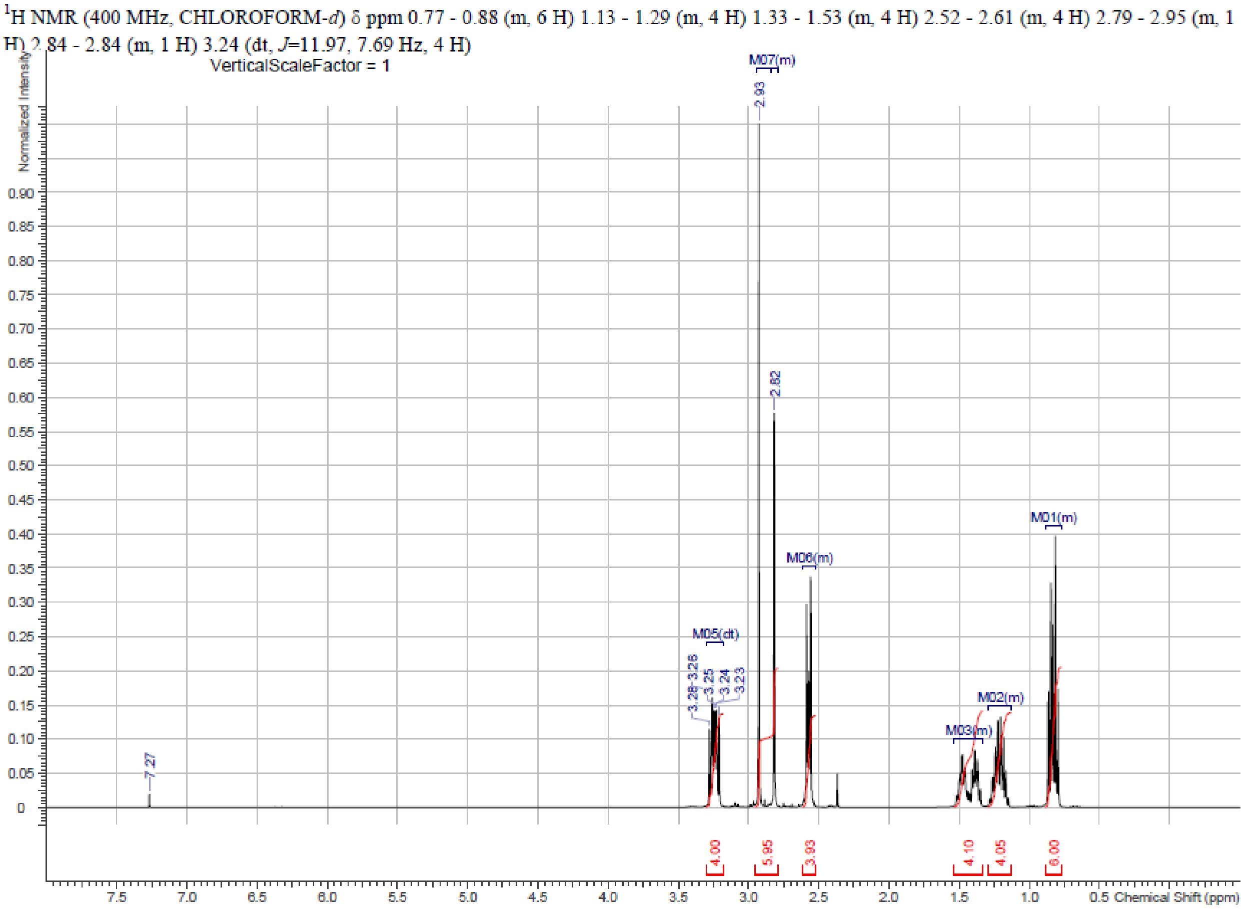

Figure S3. ^{1}H NMR spectrum of *N*,*N*'-Dimethyl-*N*,*N*'-dibutylsuccindiamide (MBSA).

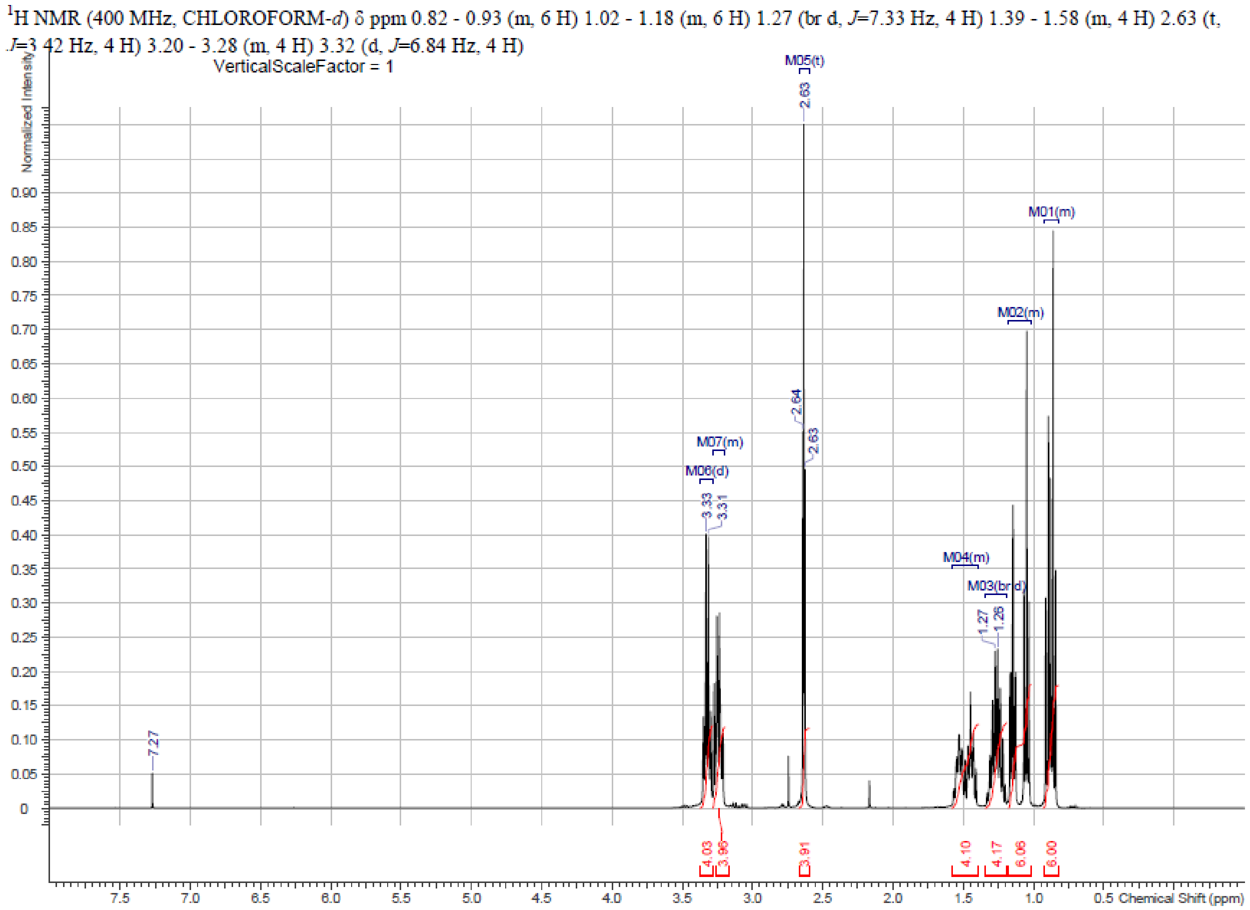

Figure S4. ¹H NMR spectrum of *N*,*N*'-Diethyl-*N*,*N*'-dibutylsuccindiamide (EBSA).

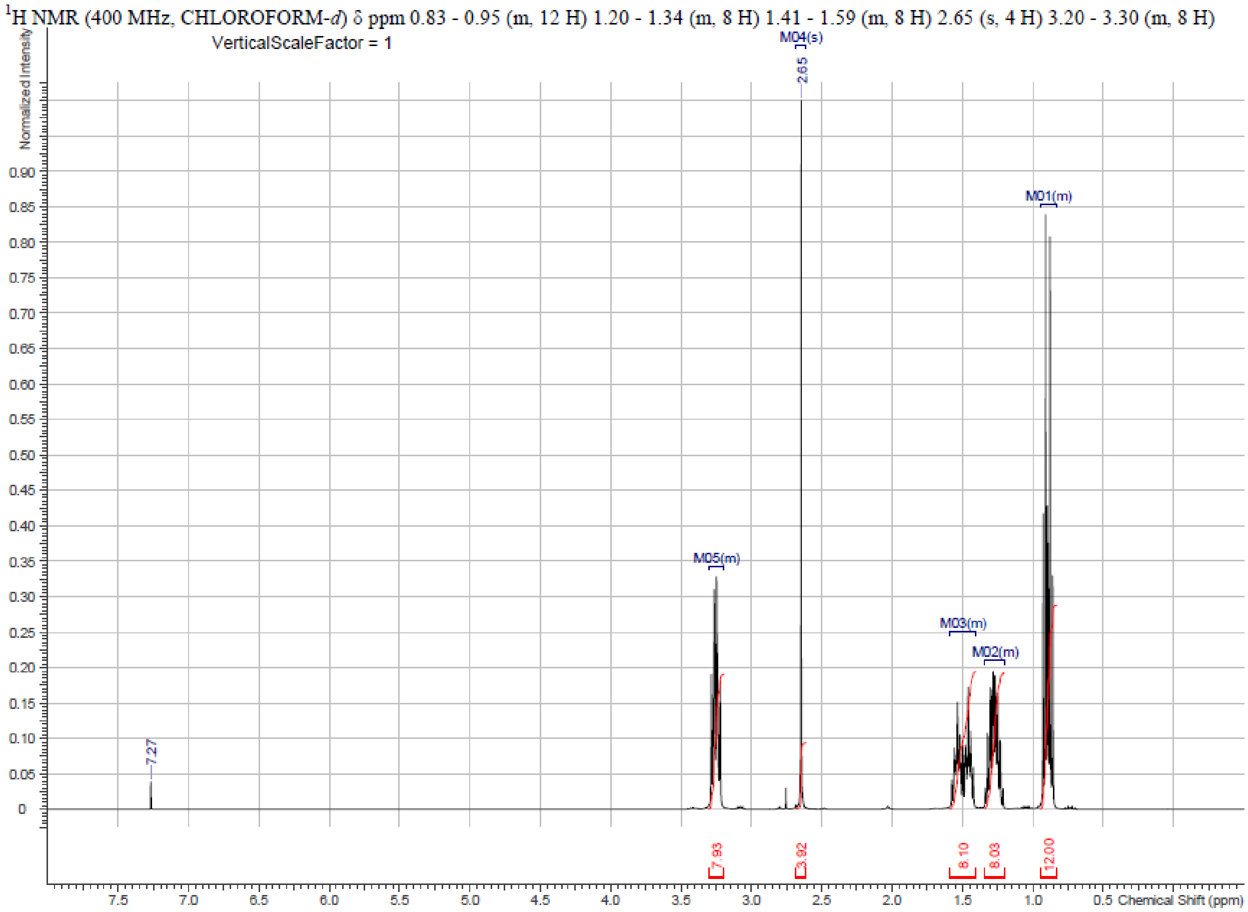

Figure S5. ¹H NMR spectrum of *N*,*N*,*N'*,*N'*-Tetrabutylsuccindiamide (TBSA).

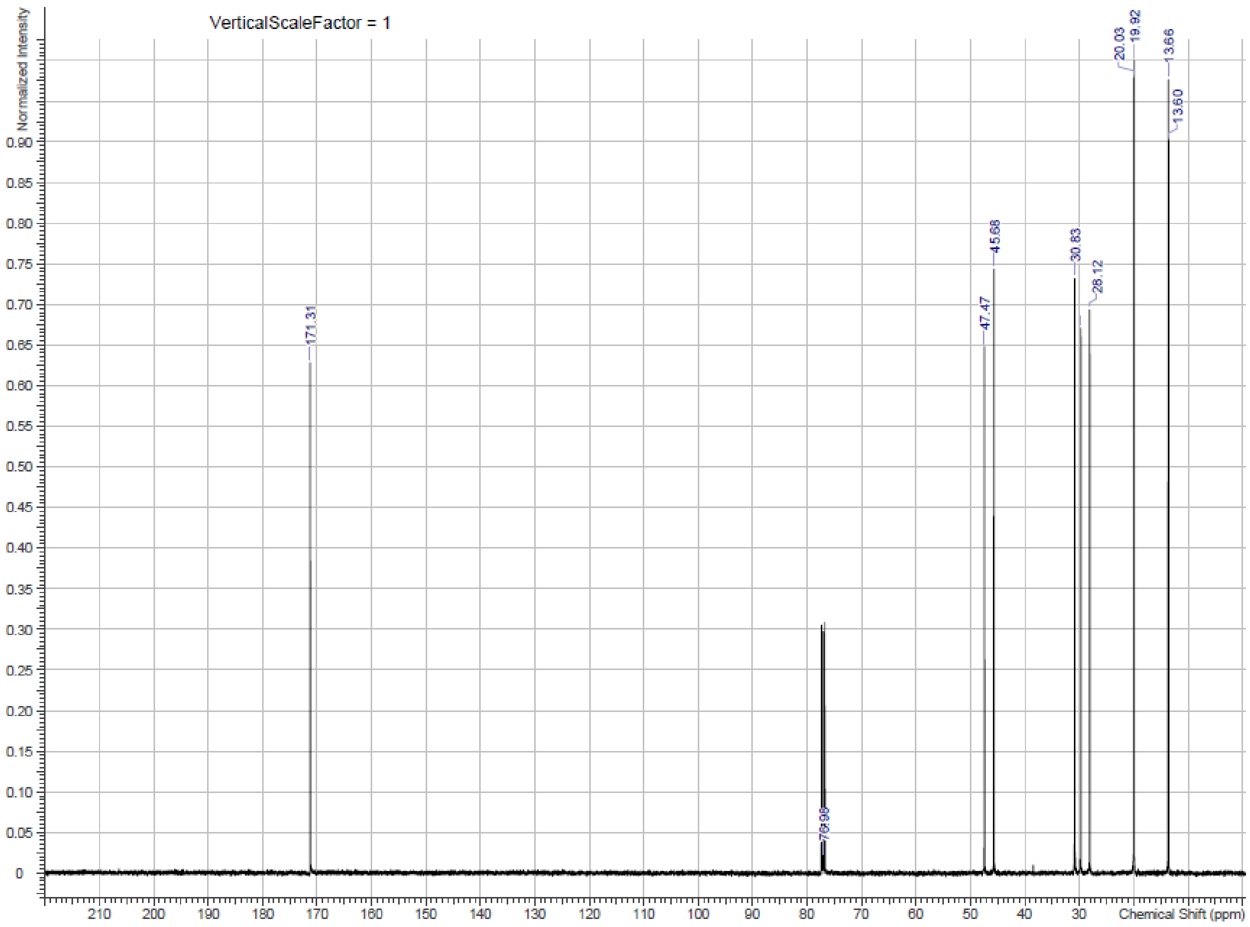

Figure S6. ^{13}C NMR spectrum of *N*,*N*'-Dimethyl-*N*,*N*'-dibutylsuccindiamide (MBSA).

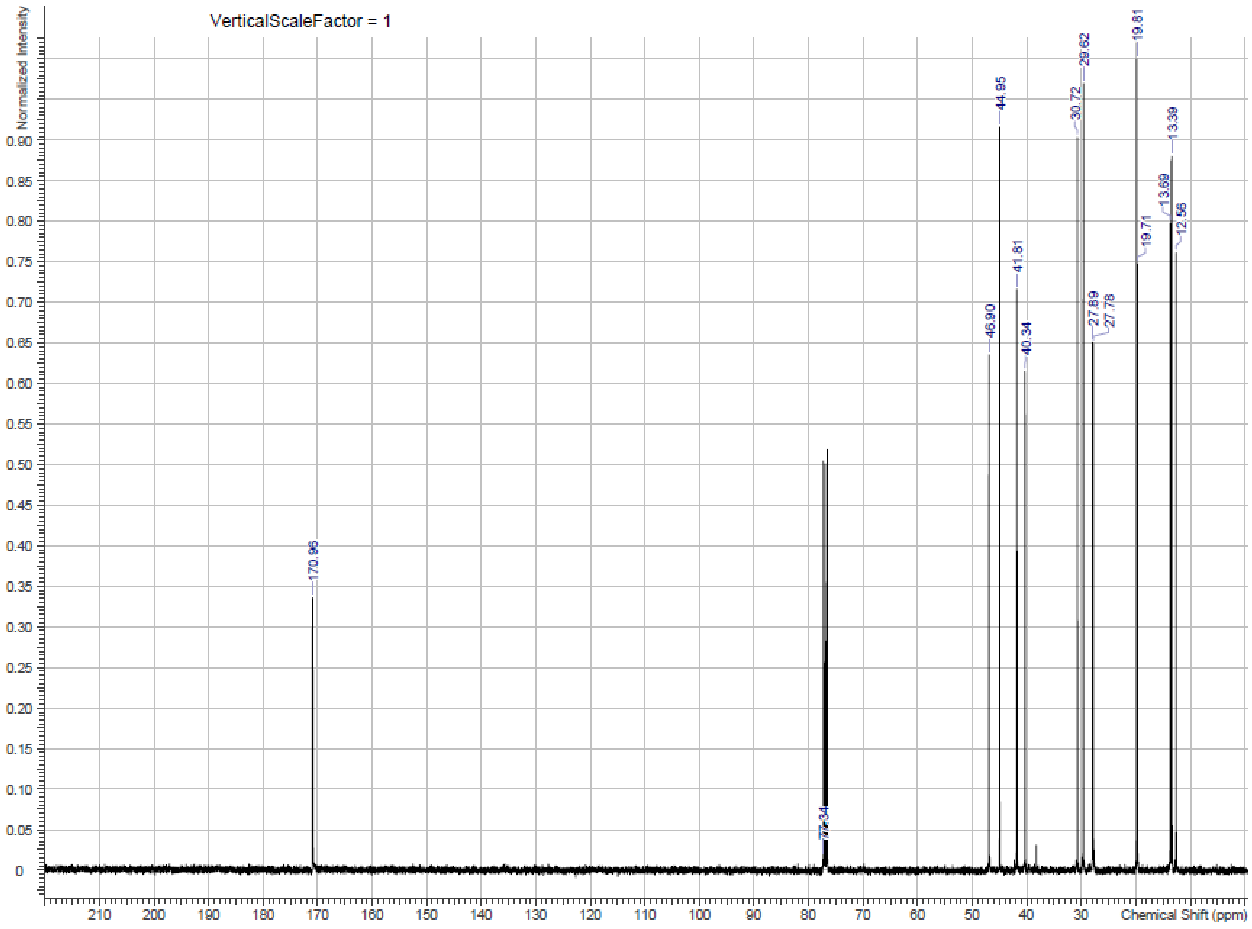

Figure S7. ^{13}C NMR spectrum of *N*,*N*’-Diethyl-*N*,*N*’-dibutylsuccindiamide (EBSA).

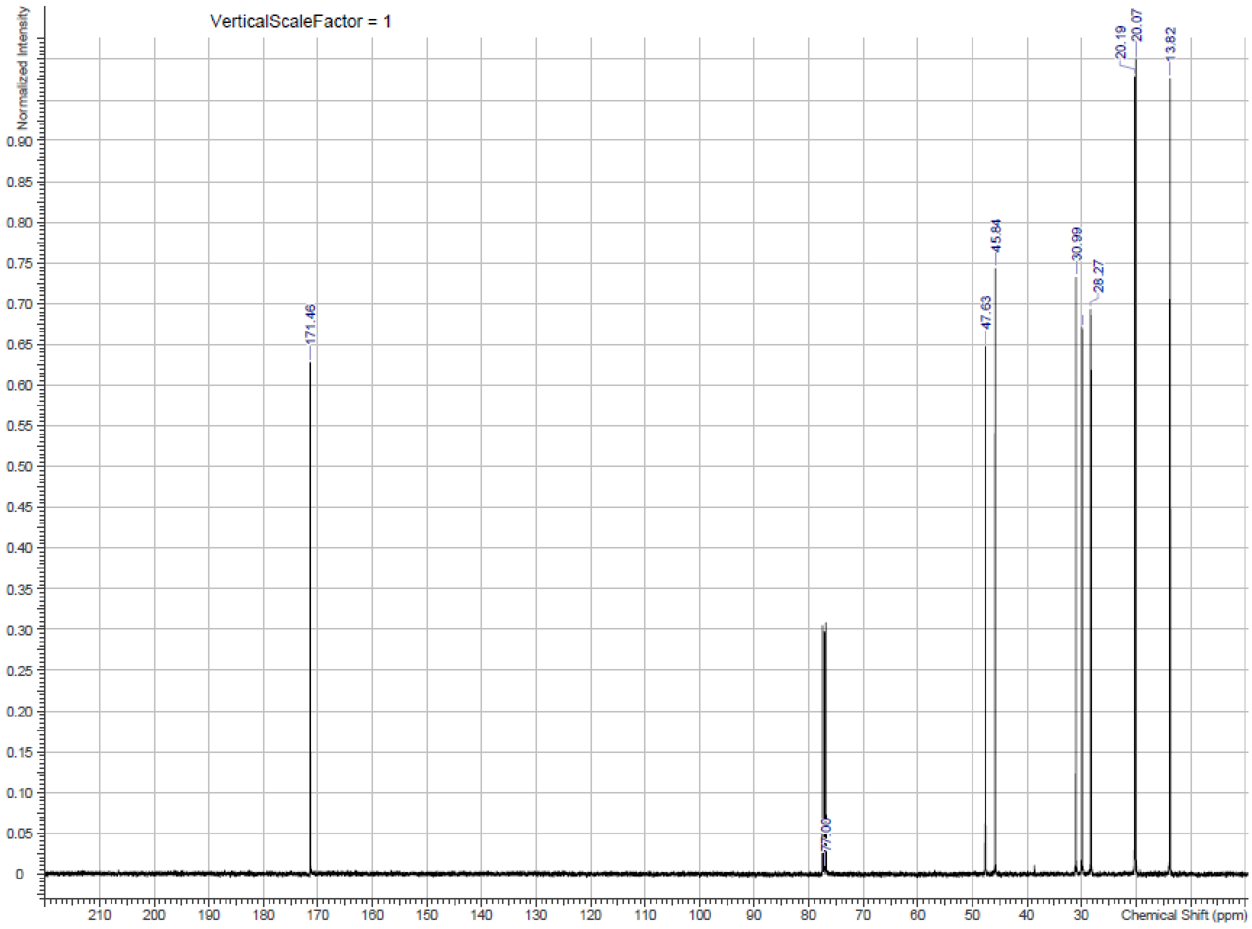

Figure S8. ^{13}C NMR spectrum of *N*,*N*,*N'*,*N'*-Tetrabutylsuccindiamide (TBSA).

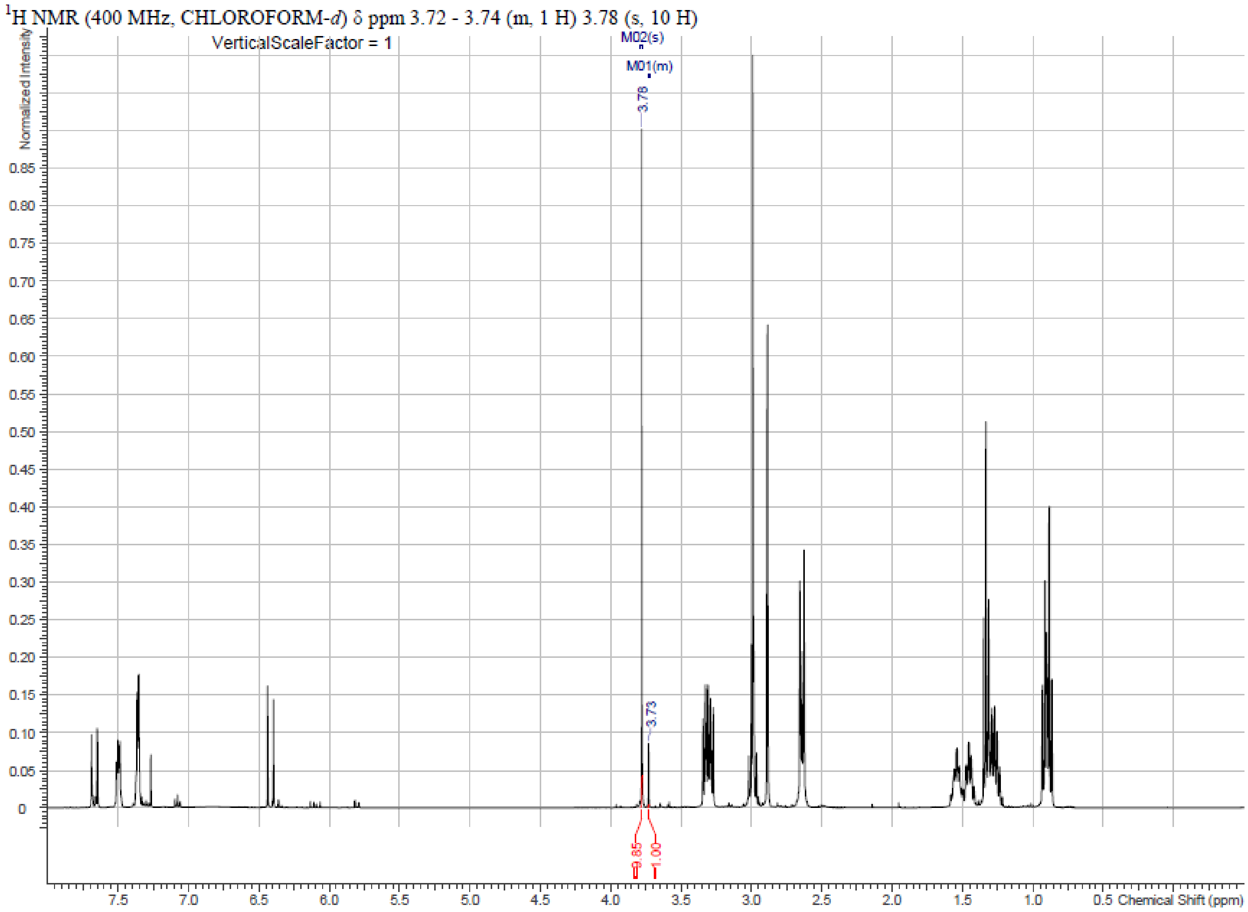

Figure S9. ^{1}H NMR spectrum of the Heck reaction mixture after 30 minutes when MBSA is used as a solvent.

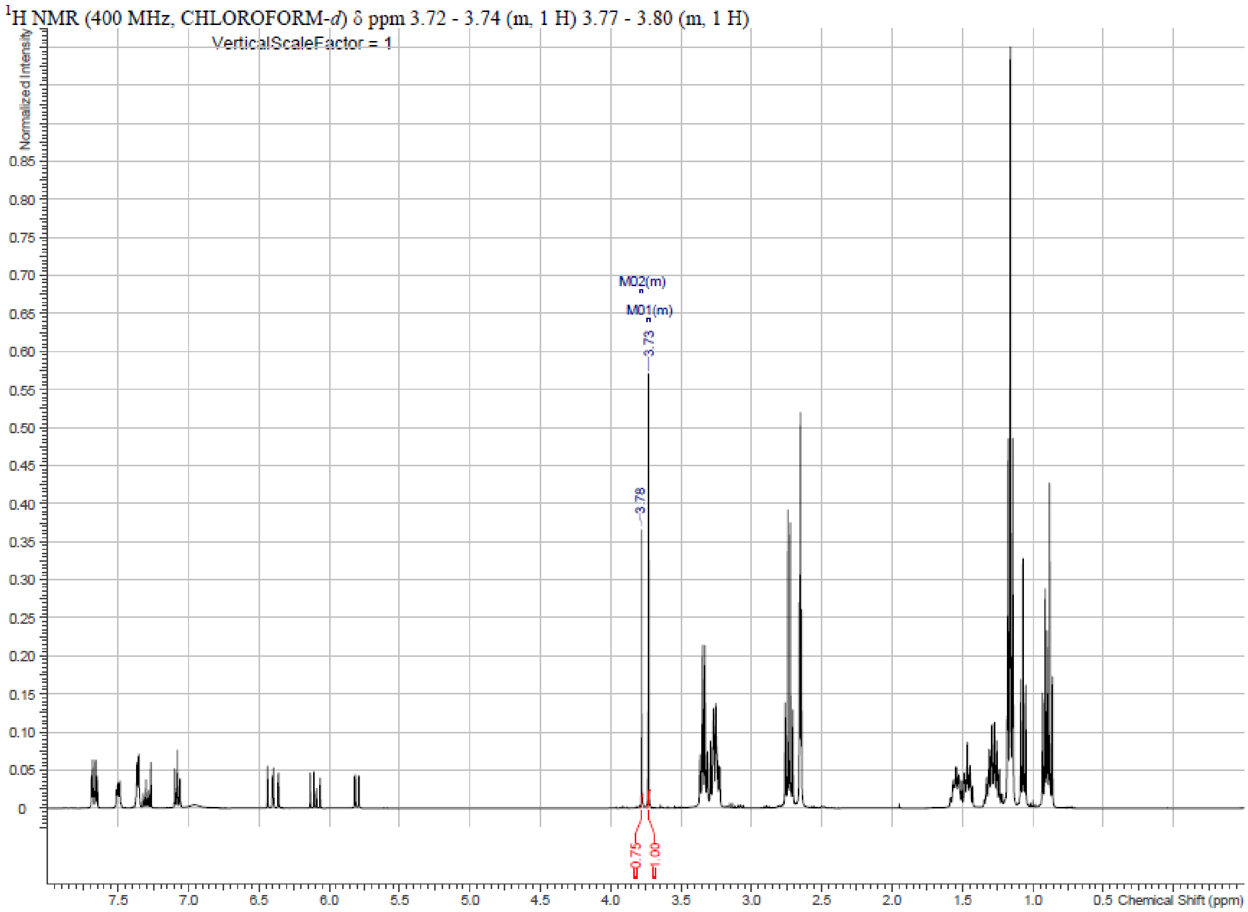

Figure S10. ^{1}H NMR spectrum of the Heck reaction mixture after 30 minutes when EBSA is used as a solvent.

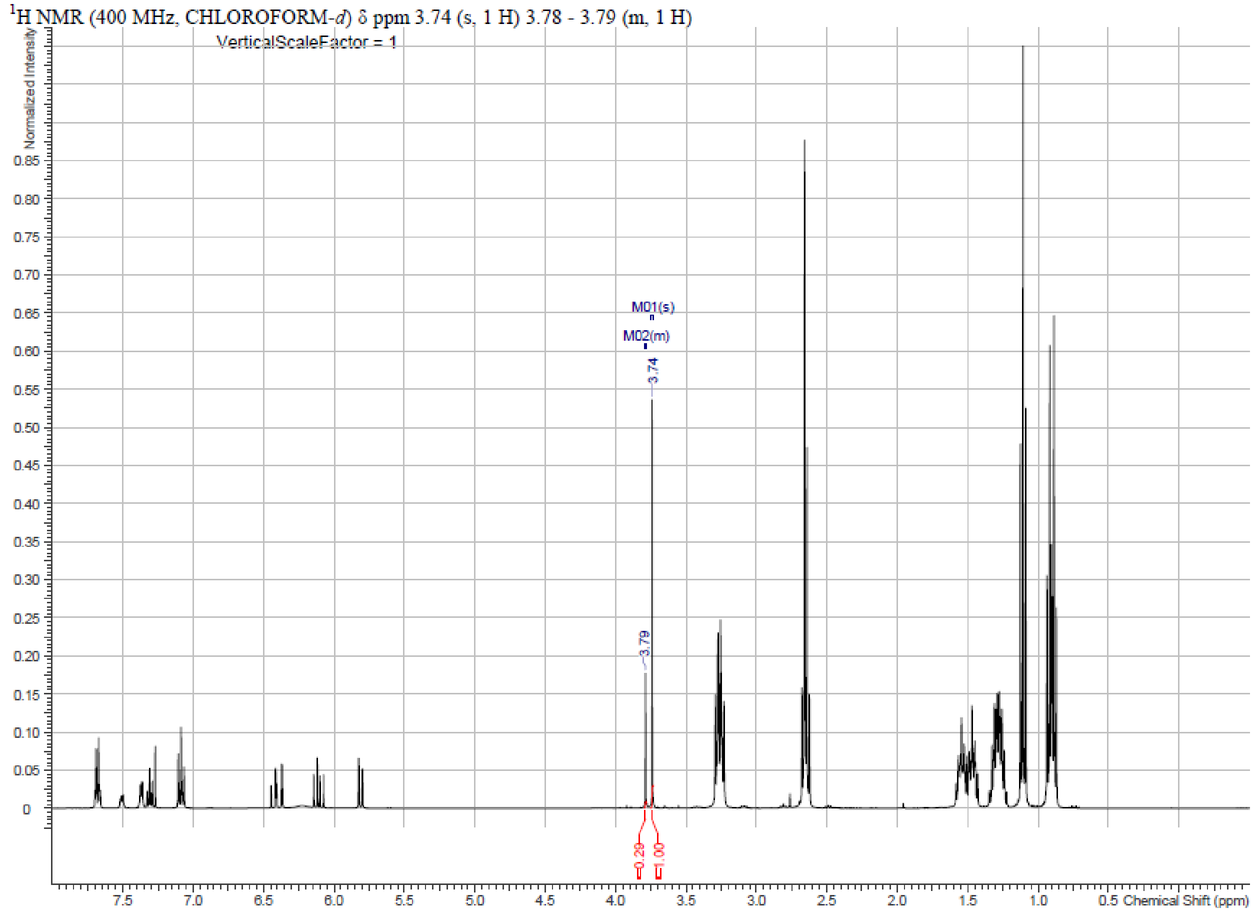

Figure S11. ^{1}H NMR spectrum of the Heck reaction mixture after 30 minutes when TBSA is used as a solvent.

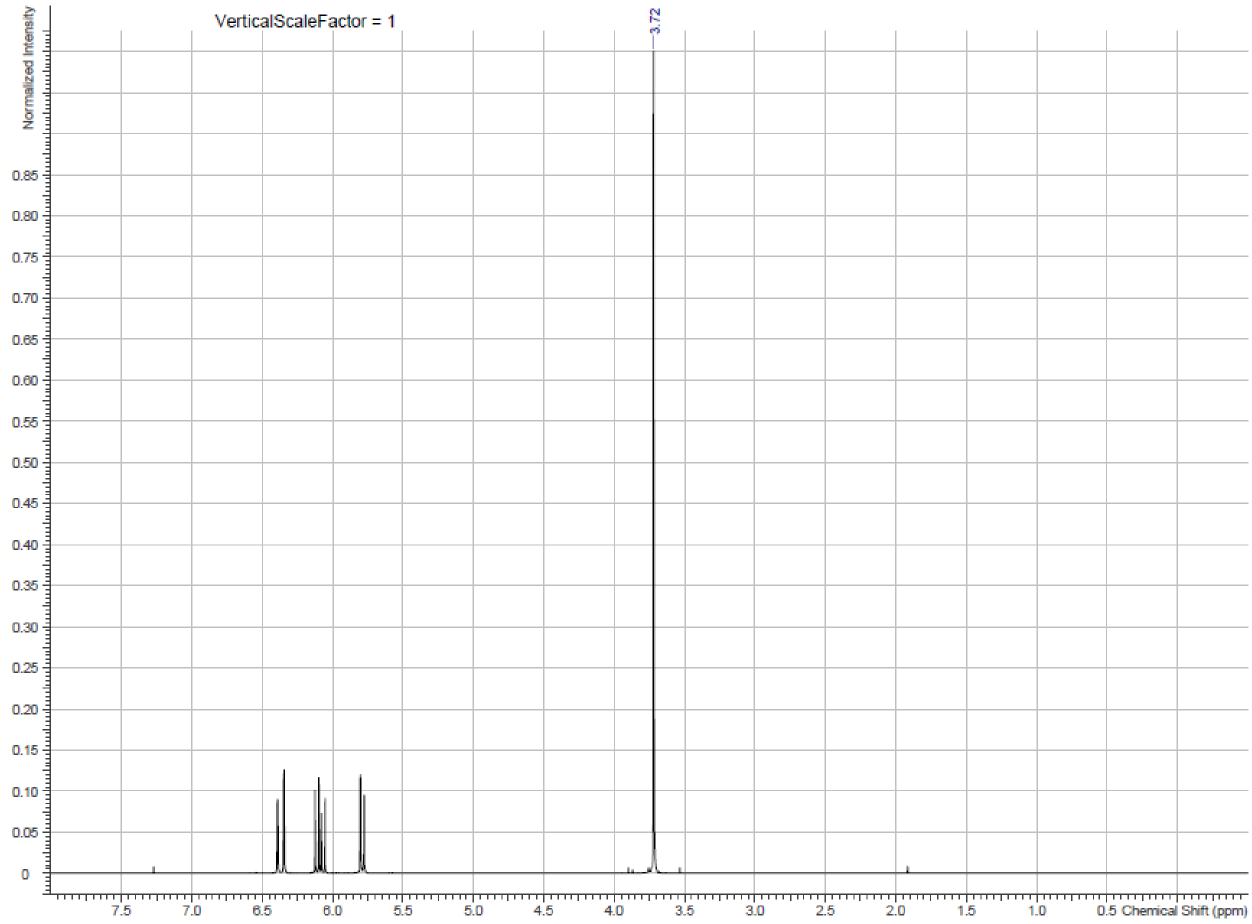

Figure S12. ^{1}H NMR spectrum of methyl acrylate (Heck reaction starting material).

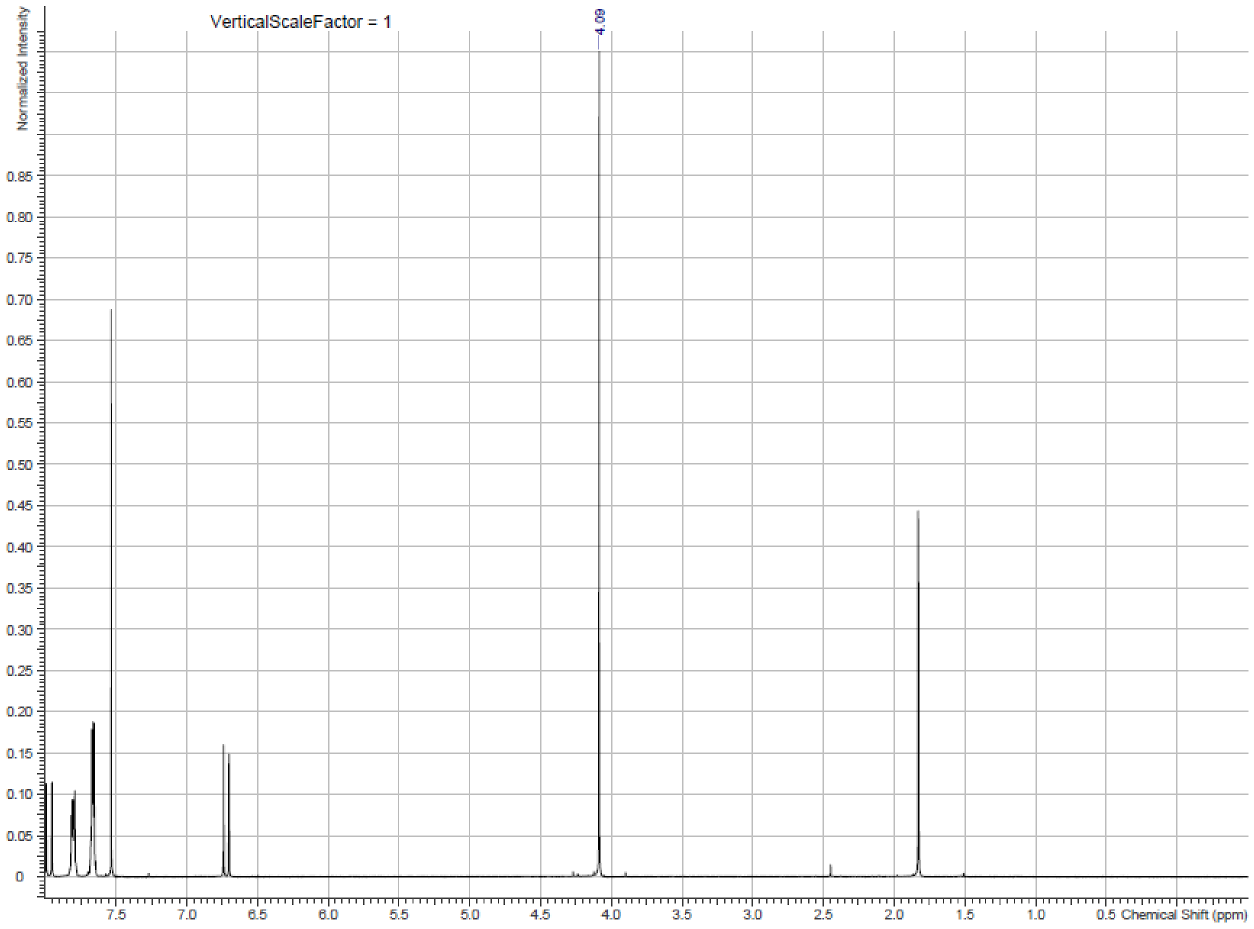

Figure S13. ^{1}H NMR spectrum of methyl cinnamate (Heck reaction product).

FT-IR spectra

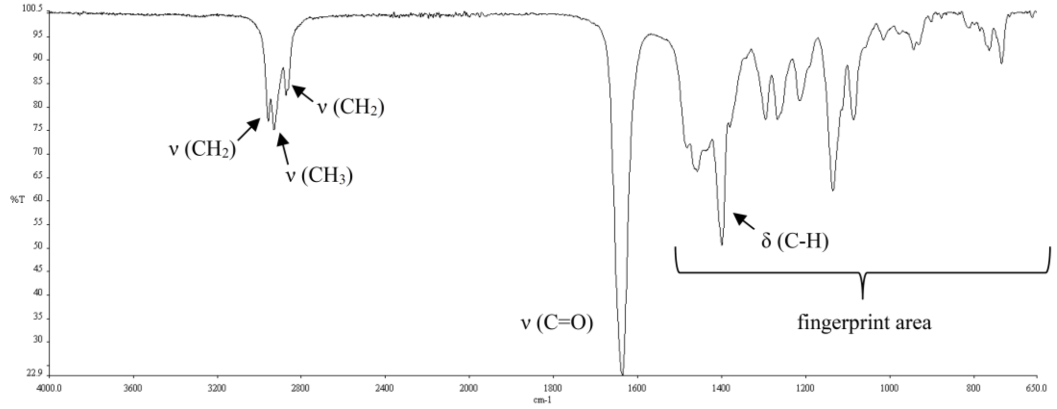

Figure S14. FT-IR spectrum of *N*,*N*’-Dimethyl-*N*,*N*’-dibutylsuccindiamide (MBSA).

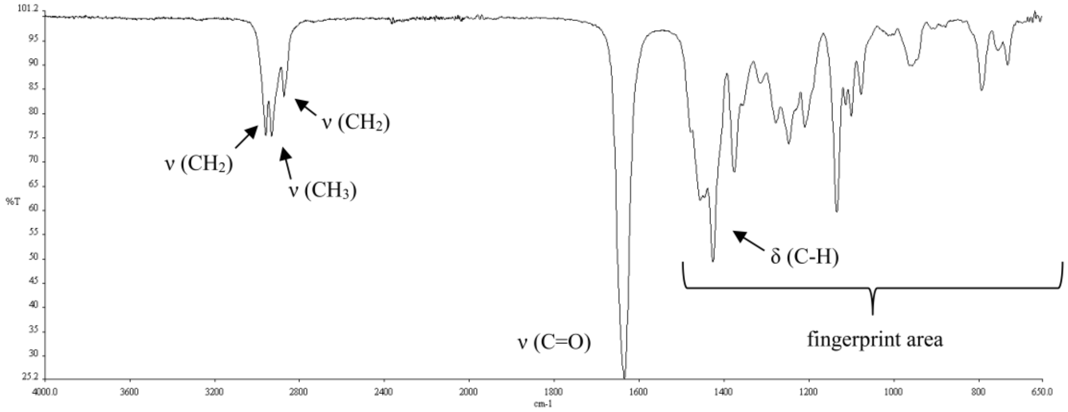

Figure S15. FT-IR spectrum of *N*,*N*’-Diethyl-*N*,*N*’-dibutylsuccindiamide (EBSA).

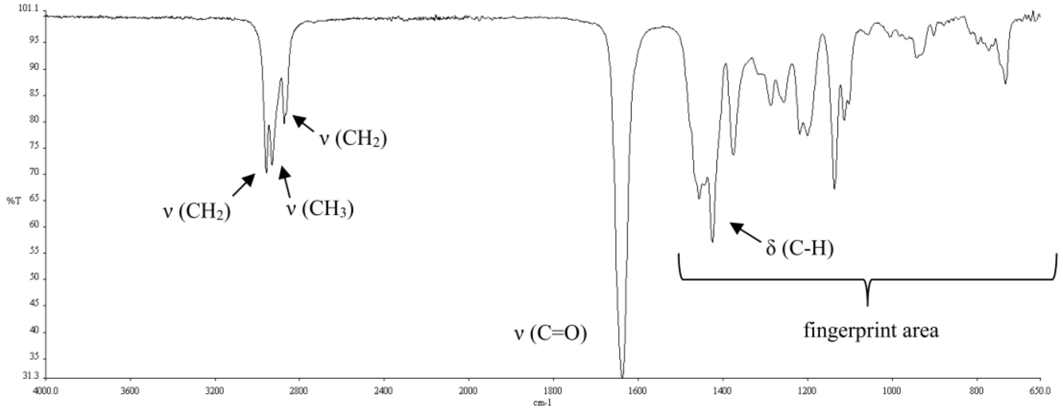

Figure S16. FT-IR spectrum of *N*,*N*,*N*’,*N*’-Tetrabutylsuccindiamide (TBSA).

DSC traces

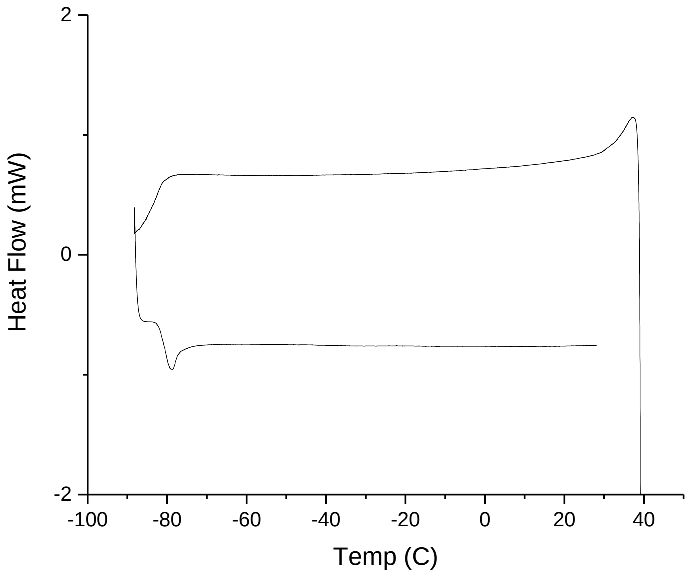

Figure S17. DSC trace of *N*,*N*'-Dimethyl-*N*,*N*'-dibutylsuccindiamide (MBSA).

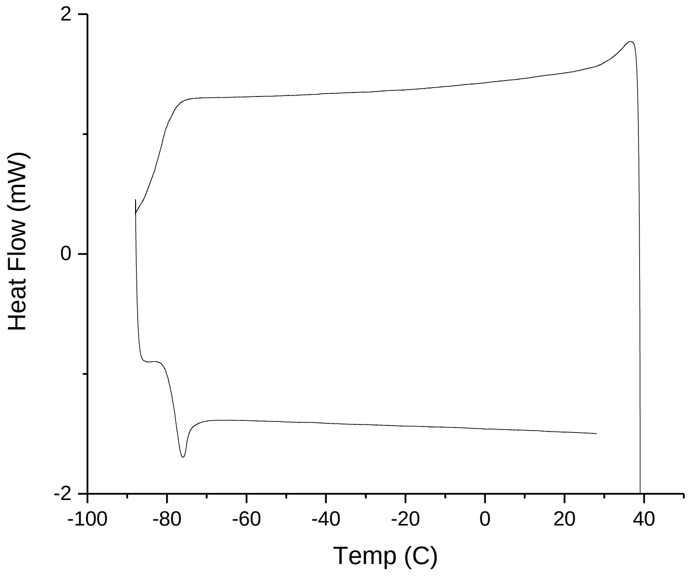

Figure S18. DSC trace of *N*,*N*'-Diethyl-*N*,*N*'-dibutylsuccindiamide (EBSA).

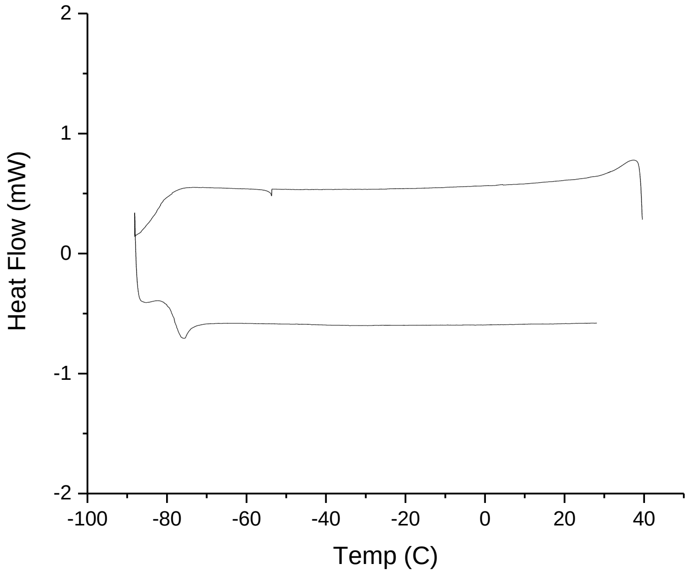

Figure S19. DSC trace of *N*,*N*,*N*',*N*'-Tetrabutylsuccindiamide (TBSA).

Porosimetry isotherms

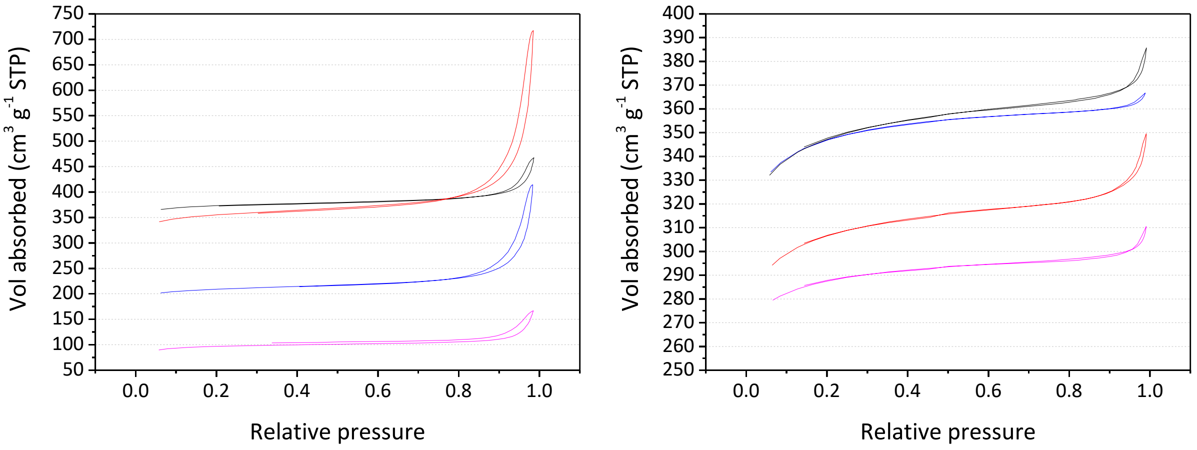

Figure S20. Isotherms for HKUST-1 (left) and ZIF8 (right) produced from DMF (black), TBSA (pink), EBSA (blue) and MBSA (red).

DTG traces

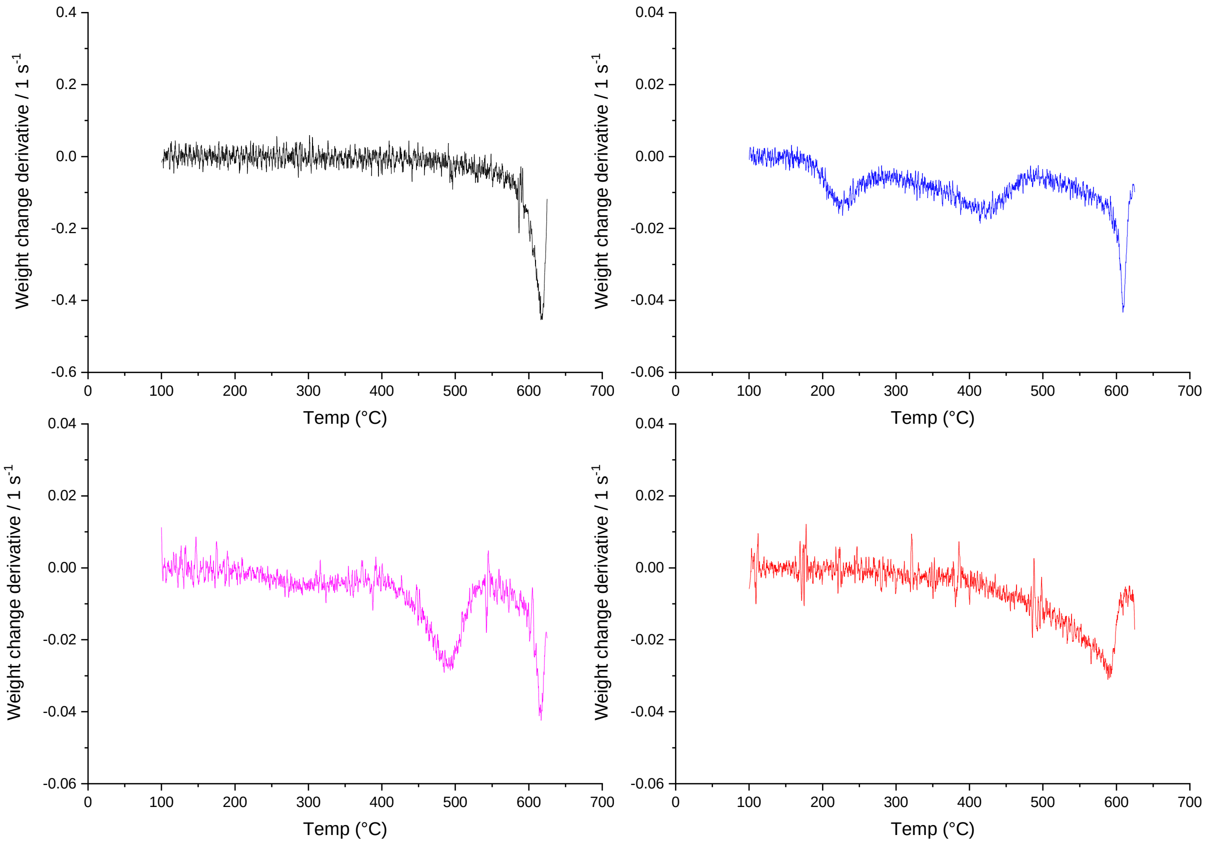

Figure S21. DTG traces of ZIF8 MOFs produced from DMF (black), TBSA (pink), EBSA (blue) and MBSA (red).

References

[1] B. van der Burg, S. van der Linden, H. Man, R. Winter, L. Jonker, B. van Vugt-Lussenburg, A. Brouwer, in *High-Throughput Screen. Methods Toxic. Test.*, John Wiley & Sons, Ltd, **2013**, pp. 519–532.

[2] E. Sonneveld, H. J. Jansen, J. A. C. Riteco, A. Brouwer, B. van der Burg, *Toxicol. Sci.* **2005**, *83*, 136–148.

[3] S. Lowell, J. E. Shields, M. A. Thomas, M. Thommes, *Characterization of Porous Solids and Powders: Surface Area, Pore Size and Density*, Springer Netherlands, **2004**.

[4] S. Park, J. O. Baker, M. E. Himmel, P. A. Parilla, D. K. Johnson, *Biotechnol. Biofuels* **2010**, *3*, 10.

[5] L. Segal, J. J. Creely, A. E. Martin, C. M. Conrad, *Text. Res. J.* **1959**, *29*, 786–794.

[6] J. Sherwood, Bio-Based Solvents for Organic Synthesis, PhD Thesis, University of York, **2013**.

[7] J. Zhang, G. B. White, M. D. Ryan, A. J. Hunt, M. J. Katz, *ACS Sustain. Chem. Eng.* **2016**, *4*, 7186–7192.

[8] "VEGA HUB – Virtual models for property Evaluation of chemicals within a Global Architecture," can be found under https://www.vegahub.eu/, **n.d.**

[9] "Danish (Q)SAR Database," can be found under http://qsar.food.dtu.dk/, **n.d.**

[10] N. Jeliazkova, M. Martinov, O. Tcheremenskaia, P. Rydberg, S. Avramova, N. Kochev, V. Jeliazkov, L. Iliev, "Toxtree – Toxtree - Toxic Hazard Estimation by decision tree approach," can be found under http://toxtree.sourceforge.net/, **n.d.**

[11] A. Worth, M. Fuart-gatnik, S. Lapenna, E. L. Piparo, R. Mostrag-szlichtyng, R. Serafimova, *The Use of Computational Methods in the Toxicological Assessment of Chemicals in Food: Current Status and Future Prospects*, **n.d.**

[12] B. van der Burg, B. Pieterse, H. Buist, G. Lewin, S. C. van der Linden, H. Man, E. Rorije, A. H. Piersma, I. Mangelsdorf, A. P. M. Wolterbeek, et al., *Reprod. Toxicol.* **2015**, *55*, 95–103.
